# The feasibility, acceptability and efficacy of an app-based intervention (the Coping Camp) in reducing stress among Chinese school adolescents: A cluster randomised controlled trial

**DOI:** 10.1371/journal.pone.0294119

**Published:** 2023-11-27

**Authors:** Xiaoyun Zhou, Sisira Edirippulige, Andrew Jones, Xuejun Bai, Anthony C. Smith, Matthew Bambling

**Affiliations:** 1 Centre for Online Health, The University of Queensland, Brisbane, Queensland, Australia; 2 Centre for Health Services Research, The University of Queensland, Brisbane, Queensland, Australia; 3 School of Public Health, The University of Queensland, Brisbane, Queensland, Australia; 4 Queensland Centre for Mental Health Research, The University of Queensland, Brisbane, Queensland, Australia; 5 Academy of Psychology and Behavior, Tianjin Normal University, Tianjin, China; 6 Centre for Innovative Medical Technology, University of Southern Denmark, Odense, Denmark; 7 Faculty of Medicine, The University of Queensland, Brisbane, Queensland, Australia; 8 School of Psychology, Australian College of Applied Professions, Brisbane, Queensland, Australia; University of Nigeria, Nsukka, NIGERIA

## Abstract

**Objectives:**

This study aimed to determine the efficacies of the Coping Camp app in reducing stress, depression, and anxiety and improving stress-coping behaviours and mental health wellbeing. Additionally, feasibility and acceptability of Coping Camp were evaluated.

**Methods:**

In this unblinded cluster RCT, 540 participants from two high schools in China were randomly assigned to the Coping Camp intervention (n = 6 classes; 275 students) or treatment as usual (n = 5 classes; 265 students) at the class level. Coping Camp was an automated self-help app, consisting of 11 sessions delivered over 11 weeks, with primary outcomes including perceived stress, depression, anxiety, stress-coping behaviours, and mental health well-being. All outcomes were assessed at baseline, post-intervention (11 weeks), and follow-up (19 weeks), with efficacy analysed using linear mixed models and feasibility/acceptability measured by a 5-point Likert scale and qualitative feedback.

**Results:**

At post-intervention and follow-up assessments, 75.4% and 81.7% of participants respectively attended. On average, participants logged in for 8.56 out of 11 sessions. Compared to the control group, the intervention group had significant reductions in levels of perceived stress (p = 0.01, d = 0.15 at T1; p < 0.001, d = 0.18 at T2), anxiety (p = 0.11; d = 0.08 at T1; p = 0.01; d = 0.13 at T2) and depression (p = 0.04, d = 0.11 at T1; p = 0.05, d = 0.10 at T2) but did not have a greater increase in stress-coping behaviours (p = 0.10 at T1; p = 0.97 at T2) or mental health wellbeing (p = 0.93 at T1; p = 0.08 at T2). The average ratings for each session were above 4, and qualitative feedback showed that most participants found the intervention to be “great,” “good,” and “useful.”

**Conclusions:**

The Coping Camp is feasible, acceptable and effective in stress management among Chinese school adolescents.

## Introduction

Adolescence is an important transitional period when adolescents experience diverse physical, psychological and social changes. The changes, including puberty, seeking independence, building peer relationships, and meeting academic and transition demands, all constitute acute or chronic stressors for adolescents. Stress, *referring to the ‘condition or feeling that results when individuals perceive that the demands of a situation exceed their personal*, *psychological or social resources’* [1], has been proven by evidence to have a fundamental impact on adolescents. For example, stress is associated with maladaptive brain development in adolescence [2] and negative mental health outcomes such as anxiety [3], depression [4] and schizophrenia [5]. Stress is also reported to be associated with decreased academic performance [6] and school dropout [7]. Also, stress is reported to be related to conduct disorders such as substance abuse [8] and juvenile delinquency [9]. In order to reduce the incidence of these mental and conduct disorders, it is imperative to provide adolescents with stress management interventions.

China has an adolescent population of 146 million and 87% are enrolled in schools [10]. According to recent reports, nearly 90% of Chinese adolescents reported that they experienced high or very high levels of stress and nearly half (45.2%) said they spent two hours or more every day on their homework [6]. Apart from academic stress, Chinese adolescents are also reported to experience stress that arises from interpersonal relationships, and family issues [11]. Students also reported that these stressors have negative impacts on their studies and emotional wellbeing [12]. However, there are no formal mental health interventions to assist Chinese high school students manage their stress. The reasons are twofold: first, the mental health workforce in China is insufficient to meet the community demand [13]. Second, there is a significant disparity in the quantity of mental health services available in urban and rural areas and across regions in China [14].

Online mental health services may provide a solution to these issues of access and quality. Globally, mental health services have been increasingly delivered via internet such as videoconference, mobile apps and portable devices. Evidence suggest that these online mental health interventions enable patients who reside in remote areas to access services while saving time and money for traveling [15]. Online self-help mental health interventions can be delivered to school students and reach a large number of users thereby reducing the demand for human professionals. In the past decade, globally, online mental health interventions have been increasingly used to manage mental health issues among adolescents and have been proven by research to be effective [16]. For example, Ahmad et al [17] delivered an 8-week online intervention that was based on mindfulness to university students and found that the intervention could significantly reduce symptoms of stress, depression and anxiety as compared with the control group. Puolakanaho et al [18] delivered an online intervention that was based on acceptance commitment therapy to secondary school students and found that the intervention could significantly reduce stress levels. A systematic review conducted by Zhou et al [16] examined the evidence on online interventions for managing mental health issues among adolescents found that among 45 studies included, 43 were conducted in westernized countries, there are only two studies which tested the efficacies of online interventions among Chinese adolescents [19, 20]. One intervention was conducted in a university and the intervention was translated from MoodGYM that was developed in Australia [20]. The other study was conducted in Hongkong district and the intervention was translated from CATCH-IT that was developed in USA [19]. Both MoodGYM and CATCH-IT were online interventions delivering modularised cognitive behavioural therapy. Research has shown that Chinese adolescents experience and cope with stress in different ways from their western counterparts due to different cultural and societal factors [21]. For example, compared with their western counterparts, Chinese adolescents are more likely to suffer from high expectations to succeed in highly competitive academic environments. In addition, Chinese adolescents frequently used internalising stress as a coping strategy. Therefore, it is important to investigate whether the use of online stress management intervention could be effective in reducing stress among Chinese adolescents. However, to our best knowledge, there are no studies that investigate the efficacy of online interventions that are developed for Chinese adolescents in managing stress. Our study therefore aimed to test whether the use of a mobile app, named Coping Camp, which was developed to meet the specific needs and preferences of Chinese adolescents in high school settings, is effective. We also aimed to test whether the use of Coping Camp was acceptable in Chinese high school settings, and whether the use of Coping Camp was effective in reducing depression and anxiety, improving mental health wellbeing, decreasing the frequency of using maladaptive coping strategies and increasing the frequency of using adaptive coping strategies among Chinese adolescents. Additionally, we also assessed whether the intervention caused harm to participants.

## Methods

We followed CONSORT-EHEALTH (Consolidated Standards of Reporting Trials of Electronic and Mobile HEalth Applications and onLine TeleHealth) [22] when reporting the results of this randomized controlled trial.

### Study design

**Fig 1:**
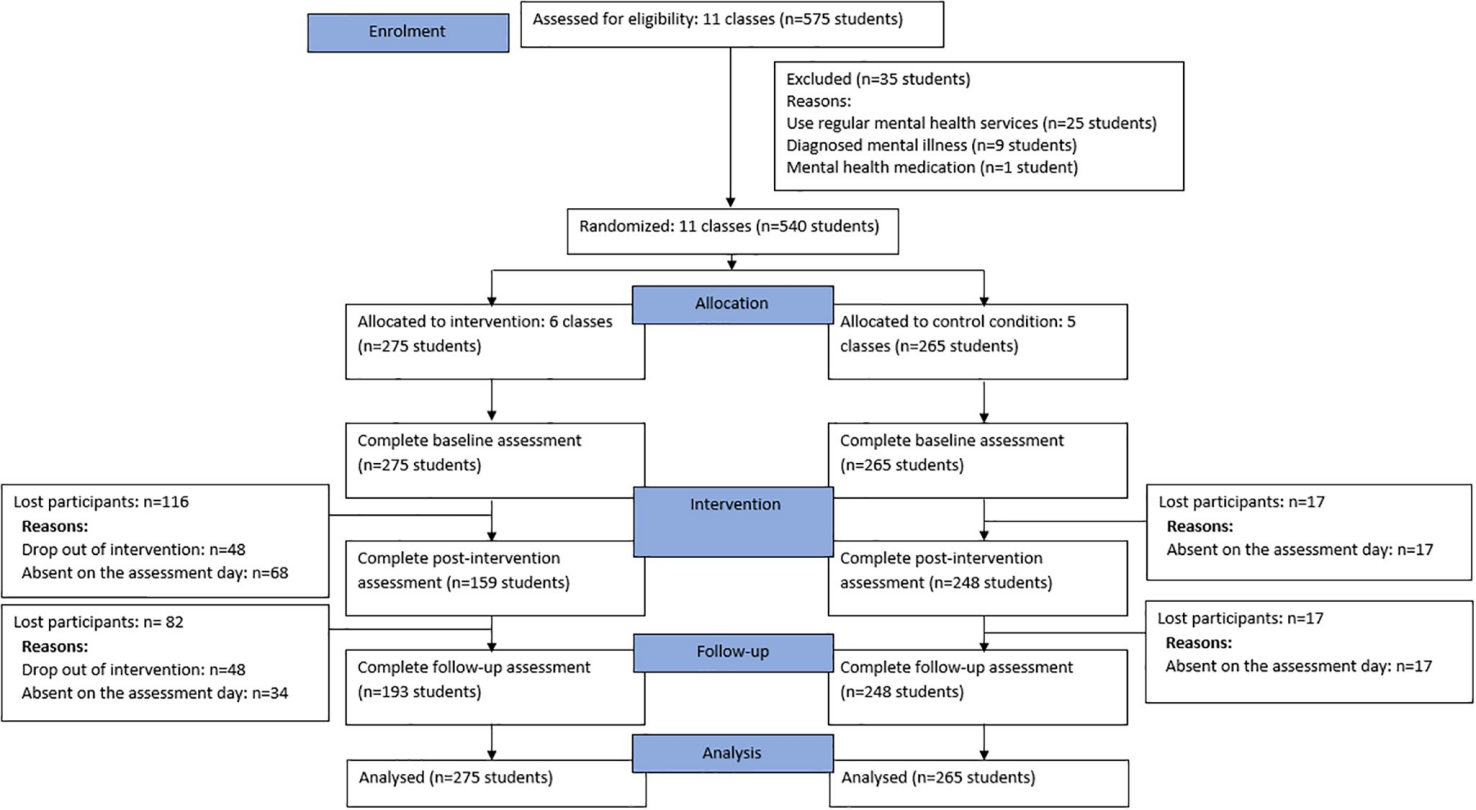
CONSORT diagram. Notes: Data was collected by gathering participating students in an auditorium and providing them with time to complete the assessment. Missing participants fall into two categories: drop-out participants and absent participants. Drop-out participants occurred when they informed the intervention implementer of their withdrawal from the study. If they did not complete the post-intervention assessment (T1), they were also missing from the follow-up assessment (T2). Absent participants were those who were not present at school on the day of assessment. Therefore, participants who were missing from the post-intervention assessment (T1) might be present at T2 and completed the follow-up assessment (T2). Therefore, the number of lost participants at T2 were counted in comparison to the baseline participants.

This study was a two-arm cluster randomised controlled trial. Participants were randomised by classes (i.e., clusters) on a 1:1 ratio to either the intervention group or the control group (see Fig 1 for CONSORT diagram). As there were 11 classes in total, one more class was allocated to intervention group to benefit more students.

### Settings

The participants were recruited from two high schools in Mianyang City of Sichuan province in China. The recruitment was conducted in September 2021. One was a private high school and the other was a public high school. The schools were anonymised due to confidentiality. The private school had a student population of around 3400. The public school had a student population of around 6000. Mianyang City is a moderately sized city located in southwest China.

### Participant recruitment

Participation in this study was voluntary. After consulting school administrators, only students who enrolled in grades 10 and 11 were available. The reason provided was that grade 12 students were preparing for the national college entrance examination, therefore, involving them was not practical. The recruitment strategies involved advertisement, posters, and announcements.

### Participant eligibility criteria

Our inclusion criteria included: (1) own a smartphone and be permitted by parents and teachers to use the smartphone; (2) be enrolled in grades 10 and 11 of high school at the time of intervention. The exclusion criteria included: (1) students should not have suicidal ideation; (2) and should not be diagnosed with a mental disorder at the time of recruitment; (3) and not be taking psychiatric medications and (4) they should not be receiving regular mental health counselling services. The eligibility criteria were assessed via self-reported questionnaires.

### Random allocation

The randomization was conducted by a research assistant who was independent of our research group. This research assistant was also blinded of which group was intervention group and which group was control group. An online randomizer (www.randomizer.org) was used to allocate classes into two groups.

### Blinding

Given the nature of the intervention, it was not possible to blind either the participants or the intervention implementer. However, the outcome assessors and the researcher who conducted the randomization were blinded to the students’ condition.

### Procedures

During the screening stage, 575 students interested in participating were invited by the first author to complete a short questionnaire to confirm their eligibility in the auditoriums at the schools. This took place on two separate weekdays during moral education class where students were engaged in non-academic activities (e.g., students watched news on TV or head teachers discussed daily affairs of the class). The participant information sheets were handed out to students to read information about the study, and they were given a chance to ask questions. After screening, 35 students were deemed ineligible (see Fig 1 for reasons) and 540 students from 11 classes were deemed eligible. These students were approached by the chief investigator, and consent forms were handed out for them and their parents to sign. All 540 students agreed to participate, and both they and their parents gave informed consent for the students to participate in the study. Intervention sessions were arranged once per week for 11 weeks during moral education classes. Each session lasted between 25–45 minutes to complete, there were no home assignments, however, the students were allowed to access the content of the app outside of intervention sessions.

### Intervention condition

Participants assigned to the intervention group (IG) received a QR code to download the Coping Camp application, which had two versions: Android and IOS versions to suit different students. After downloading the Coping Camp, students were required to create a personal account, which they must use to log into the Coping Camp.

The intervention was based on Stress Inoculation Training (SIT) [23], a type of Cognitive Behavioural Therapy aimed at reducing stress. Although SIT has been frequently used to manage PTSD among veterans, it has been widely adopted in preventive and treatment interventions for stress management among nurses [24], teachers [25], and adolescents [26]. In recent years, SIT has been delivered via virtual reality technology and mobile phones and demonstrated potential efficacies [27, 28]. The standard SIT comprises three phases: the educational phase, skills training phase, and application phase. We modified the SIT to suit the Chinese culture and our adolescent population based on our previous studies with Chinese high school students and teachers [29]. Then, we invited Chinese academics and professionals to review the content of our SIT intervention, minor changes were made according to their comments. As a result, the modified SIT had 11 sessions. Modified SIT consisted same phases as standard SIT, the skill training phase included somatic skills (progressive relaxation and mindfulness), cognitive restructuring skills and behavioural skills (goal setting, time management, and problem-solving skills) and interpersonal skills. More details of the intervention can be found in our protocol: dx.doi.org/10.17504/protocols.io.n92ldmz69l5b/v1 [PROTOCOL DOI]. An app named Coping Camp was developed based on the modified contents of SIT. The app was in Chinese language and programmed by a Chinese company. To assess the initial feasibility and acceptability, we conducted a pilot test of the Coping Camp app among students prior to this trial, and bugs were identified and fixed.

**Fig 2:**
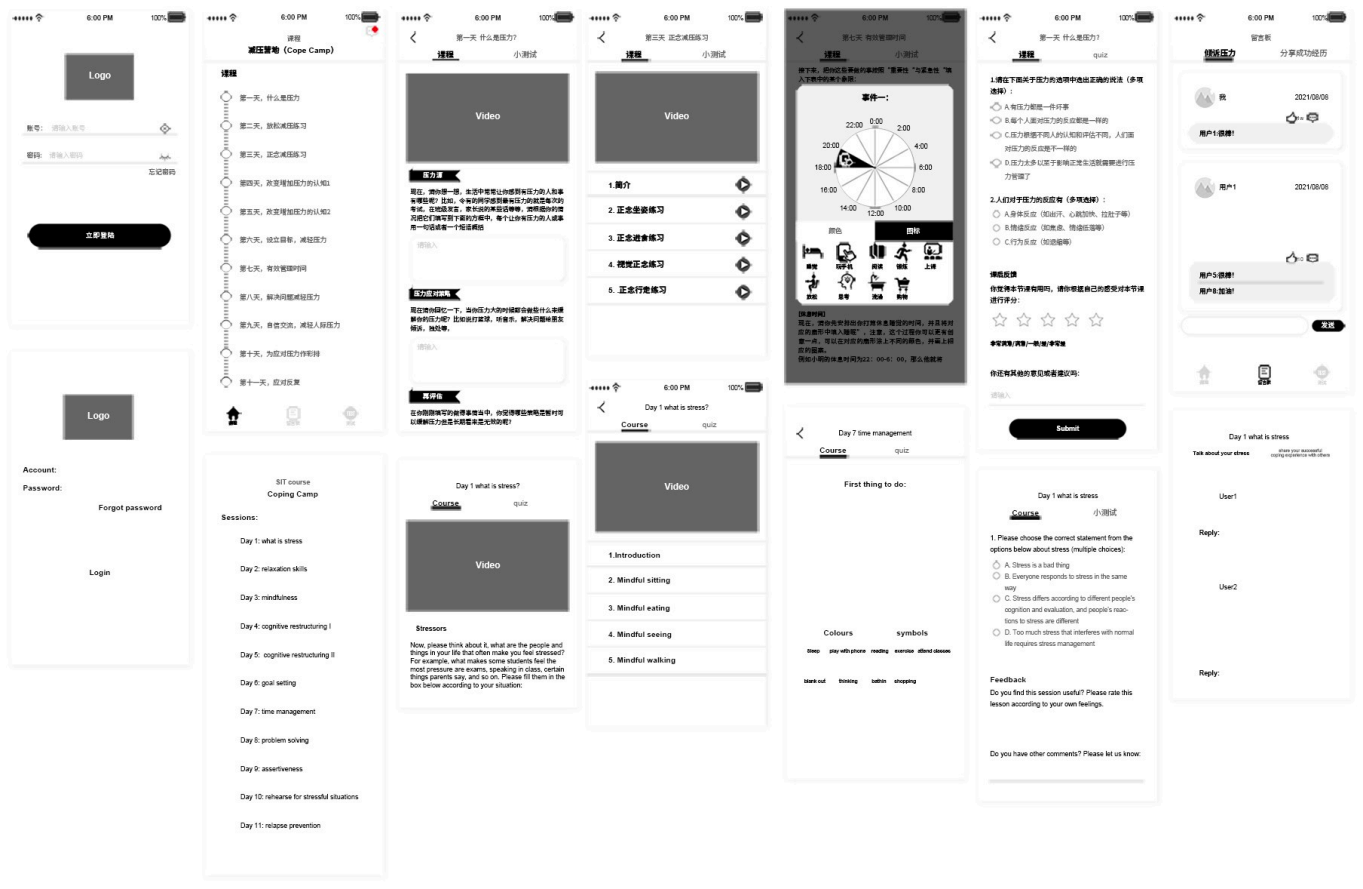
Example pages of the app prototype.

The Coping Camp comprised 11 sessions (Fig 2), notification functions, three assessment and a discussion board. The 11 sessions were locked, and students could only access the next session after completing the previous one. The three assessments were also locked and only open at the time of each assessment. The discussion board was open during the intervention, and any participant could post or reply to other posts on the discussion board. The discussion board had two options, one named "Tell Us About Your Stress" and the other named "Share About Your Successful Coping Experiences." The aims of the discussion board were to provide a peer-support forum for students to discuss their stress, learn from and support each other, while still limiting the content to stress and coping. The discussion board was monitored by the chief investigator (XZ). The moderator only interacted with students on the discussion board under two circumstances: (1) when a question was directly asked to be answered by the moderator, and (2) when posts or replies were consulting other participants, the moderator would delete these posts or replies.

### Control group

Participants who were allocated to a control group (CG) attended their regular moral education classes as arranged by the schools.

### Measures

All the participants in the intervention or control group were assessed at three timepoints: baseline (T0; prior to intervention), post-intervention (T1; 11 weeks after the baseline), and follow-up (T2; 19 weeks after the baseline). The measures included demographic variables and study outcomes. Demographic variables were collected at baseline. We collected gender, year of birth, class, grade, name of the school, residence (rural or urban), and guardians for demographic information. Study outcomes were introduced in below sections. Data were collected via online questionnaires in the app and in the classrooms.

#### Perceived stress

The primary outcome was perceived stress which was assessed by Perceived Stress Scale-10 Items (PSS-10). The PSS-10 is a self-reported scale comprising ten items used to evaluate respondents’ global level of perceived stress [30]. Respondents were asked to rate the frequency of certain feelings they experienced over the past month on a 5-point Likert Scale (1 = never, 2 = rarely, 3 = sometimes, 4 = often, 5 = always). The PSS-10 has been translated in Chinese and shown acceptable reliability and validity in both adults [31] and adolescents [32].

#### Depression and anxiety

The Depression, Anxiety and Stress Scale (DASS-21) was used to measure symptoms of anxiety and depression as secondary outcomes. The DASS-21 is a 21-item scale that rates the degree to which each statement applies to participants over the past week on a four-point Likert scale from 0 to 3. Total scores range from 0 to 42, with higher scores indicating more severe symptoms. Subscale scores were also utilized in the study. The DASS-21 has been validated in Chinese adolescent populations and has satisfactory psychometric properties [33].

#### Mental health wellbeing

Mental health wellbeing was measured using the four-item self-report instrument called the Outcome Rating Scale (ORS-4). The ORS-4 assesses the perceived change in three areas of client functioning: individual (or symptomatic) functioning, interpersonal relationships, and social role performance (work adjustment, quality of life). The ORS-4 has been found to have adequate reliability and validity [34], and the Chinese version of the ORS-4 has been recently validated and shown to be a reliable and valid measure for Chinese sub-clinical clients [35].

#### Coping behaviours

Stress coping behaviours were measured using the short form of the Coping Inventory for Stressful Situations (CISS-SFC), which is a 21-item scale that measures task-oriented coping, emotion-oriented coping, and avoidance-oriented coping. Task-oriented coping was regarded as maladaptive coping, and emotion- and avoidance-oriented coping were regarded as maladaptive coping [36, 37]. The CISS-SFC has been validated and shown to be reliable among Chinese university students after being translated into Chinese [38].

#### Negative events

To measure the negative effects of the intervention on a universal sample of school students, we defined negative events as any significant unfavorable changes perceived by participants in their life and study, rather than solely as significant changes in their mental condition during the intervention period. Serious negative events were defined as mortality, hospitalization, suicide, or suicide attempts. Short questionnaires with open-ended questions were used to measure negative events. Data related to negative events was collected at the end of the intervention period (T1).

#### Acceptability

Acceptability of the intervention was measured through feedbacks at the end of each session which was assessed using a short satisfaction survey. The survey had two questions: the first was a quantitative question asking participants to rate the usefulness of Coping Camp on a 5-point scale, with 1 indicating "not useful at all" and 5 indicating "most useful." The second question was qualitative, asking participants to provide comments about each session.

### Sample size calculation

According to a meta-analysis on internet interventions on stress reduction [39], the overall mean effect size for stress at post-test was Cohen’s d = 0.43 (95% CI 0.31–0.54). Small but significant effects were also found for depression (Cohen’s d = 0.34, 95% CI 0.21–0.48) and anxiety (Cohen’s d = 0.32, 95% CI 0.17–0.47). Therefore, effect sizes of d = 0.43 for perceived stress, d = 0.34 for depression, and d = 0.32 for anxiety were chosen. For a cluster randomized trial assuming the same values as for the original design, allowing for variation in cluster size up to a coefficient of variation of 5, an intra-cluster correlation up to 0.25, an individual autocorrelation as low as 0.5, and up to 10% drop out in each cluster, 5 clusters per group with an average size of 22 were needed to detect this effect (d = 0.43) with a power (1− β) of 0.80 at an alpha of 0.05, resulting in a total of 220 participants for all clusters across both groups.

### Statistical analysis

Statistical analyses were conducted using software R version 4.2.2 (the R Foundation for Statistical Computing, Vienna, Austria). The accepted level of significance for this study was set at 95%. To determine baseline differences between the intervention group and control group, independent t-tests were performed on continuous baseline variables (e.g., age, PSS-10, DASS-21, ORS-4, CISS-SFC, MER), and Chi-squared tests were performed on categorical or nominal variables (e.g., gender, home location, guardian, etc.). To evaluate the efficacy of the intervention, intention-to-treat (ITT) analyses were conducted separately for each outcome [40]. The ITT analysis included data from the entire randomized sample. A repeated measures design with a linear mixed model (LMM) was used to investigate the effects of time and groups on outcome variables. The LMM was performed with time, group, time*group, gender, school, grade, guardian, and residence treated as fixed effects, while students and classes were treated as random effects. Covariates that are not significant will be removed from the model, therefore the main analyses will only include significant covariates. Cluster effects were examined by using regression model, where cluster (i.e., class) was used as a fixed effect, for each outcome at T1 and T2 separately, and the analysis results suggested that there were no significant cluster effects. In each linear mixed model, the school effects were assessed as a fixed effect. Upon analysis, these effects were found to be statistically insignificant in all models. Consequently, the final models did not incorporate the school effects. Additionally, given the large endpoints, p values (two-sided) will be adjusted using the Benjamini-Hochberg method to control for false discovery rate [41]. Last, we used raw scores rather than adjusting them for baseline difference in LMMs based on following reasons: for each baseline covariate presented in the table, we re-evaluated the p-values, taking into account adjustments for clustering, by employing a mixed effects model. This adjustment incorporated the treatment group as a fixed effect and the cluster as a random effect. Post-adjustment, only ORS-4 was statistically significant. All other variables were non-significant with the same randomization. This is likely to be attributed to the potential for false positives. Additionally, since the linear mixed model (LMM) introduces a random intercept for each class, baseline variations are expected to exert minimal influence on the results. Furthermore, additional analysis was performed for ORS-4, using the difference to baseline, and were similar to those of using the raw scores.

### Missing participants and missing data handling

Data was collected by gathering participating students in an auditorium and providing them with time to complete the assessment. Therefore, missing participants fall into two categories: drop-out participants and absent participants. Drop-out participants occurred when they informed the intervention implementer of their withdrawal from the study. Except for one class (n = 48) students who withdrew from the study due to one subject teacher believed that the students should spend more time studying, after two weeks from the start of the intervention, there were no more students or classes who withdrew. In this case, they did not complete the post-intervention assessment (T1) or follow-up assessment (T2). Absent participants also occurred when they were not present at school on the day of assessment. Therefore, participants who were missing from the post-intervention assessment (T1) might be present at T2 and completed the follow-up assessment (T2). Therefore, the number of lost participants at T2 were counted in comparison to the baseline participants.

Missing data occurred in two different scenarios. The first scenario was when participants dropped out of the intervention or were not present on the day of assessment. At time point 1, there were 133 missing participants. Of those, 116 were from the intervention group, with 48 dropping out of the intervention and 68 not being present on the assessment day, while 17 were from the control group. At time point 2, there were 99 missing participants, with 17 from the control group and 82 from the intervention group. For the missing participants from the intervention group at time point 2, 48 had already dropped out at time point 1, and 34 were not present on the day of assessment at time point 2. Even though some participants did not complete all the time points, their data was still used in the analyses. The participants who dropped out did not differ significantly from those who remained in the trial regarding participant characteristics and study outcomes.

The second scenario was when a single participant skipped questions on a questionnaire such as PSS-10 or DASS-21. In this case, the missing data points were coded as NA and treated as missing in our analysis in R. Any participants with missing data for given outcomes were removed from the analysis of those outcomes. Missing data were not significantly associated with participants’ characteristics or study outcomes. To test the robustness of our results, we compared LMM results of including only those with complete data with results of including all the participants and found that the two results remained almost unchanged.

### Ethical review

Ethics approvals were obtained from two institutions: the Human Ethics Office at The University of Queensland in Australia (approval number: 2021/HE000791) and the Research Ethics Office at Tianjin Normal University in China (approval number: 2021041901). Informed written consent forms were obtained offline from both the participants and their guardians as most of the participants were under the age of 18 at the time of the trial. Participation was voluntary, and all students were informed that their answers would remain confidential. Only the first author had access to identifiable information during and after data collection. All participants were anonymized prior to data analysis.

### Trial registration

This randomised controlled trial was registered via Australian New Zealand Clinical Trials Registry (ANZCTR). Registration number: 380316. Link to the trial registration: https://www.anzctr.org.au/Trial/Registration/TrialReview.aspx?id=380316&isReview=true.

## Results

### Study participation

A flowchart of the study participants is shown in Fig 1. At the time of screening assessment in 11 school classes, a total of 613 students were present. Among them, 575 students (93.8%) agreed to participate. Of these, 540 students (88.1%) meet the inclusion criteria and participated in the study. Six classes consisting of 275 students were randomly allocated to the intervention group and 5 classes consisting of 265 students were allocated to the control group. Post-intervention assessment was completed in 159 participants (57.8%) in the intervention group and 248 participants (93.6%) in the control group. Follow up assessment was completed in 193 participants (70.2%) in the intervention group and 248 participants (93.6%) in the control group.

### Sample characteristics

Table 1 presents the baseline characteristics for the study population. Baseline differences were found between intervention and control group participants for the following variables: the avoidance subscale of the CISS-SFC (t = -2.347, p = 0.019) and ORS (t = 3.123, p = 0.002), indicating that the intervention group had lower scores on avoidance coping skills (mean 19 vs. 20) and higher levels of mental health well-being (mean 26 vs. 24) than the control group at the start of the study. There were no other statistically significant differences found for demographic or outcome variables.

**Table 1:** Demographic and baseline characteristics of trial participants (N = 540).

	Intervention, N = 275	Control, N = 265	P value
**Age (years), mean (SD)**	16.76 (0.76)	16.84 (0.73)	0.27
**Sex, n (%)**			0.51
Female	124 (45)	111 (42)	
Male	150 (55)	153 (58)	
Unknown	1	1	
**School, n (%)**			0.07
Private school	133 (48)	150 (57)	
Public school	142 (52)	115 (43)	
**Grade, n (%)**			0.18
Grade 10	135 (49)	114 (43)	
Grade 11	140 (51)	151 (57)	
**Residence, n (%)**			0.51
Urban area	205 (88)	240 (91)	
Rural area	27 (12)	25 (9.4)	
Unknown	43	0	
**Guardian, n (%)**			0.31
Parents	267 (98)	259 (98)	
Relatives	3 (1.1)	1 (0.4)	
Others	2 (0.7)	5 (1.9)	
Unknown	3	0	
**PSS-10, mean (SD)**	18 (6)	17 (6)	0.14*
**DASS-21, mean (SD)**	16 (10)	17 (9)	0.77
Unknown	1	1	
Depression subscale	4.4 (3.7)	4.5 (3.6)	
Anxiety subscale	5.3 (3.6)	5.3 (3.6)	
Stress subscale	6.7 (3.6)	6.9 (3.3)	
**CISS-SFC, mean (SD)**	62 (13)	63 (11)	0.33
Unknown	1	6	
Emotion subscale	20.6 (5.5)	20.8 (5.3)	
Task subscale	23 (6)	22 (6)	
Avoidance subscale	19 (6)	20 (6)	
**ORS-4, mean (SD)**	26 (8)	24 (7)	0.03*
Unknown	3	6	

Note:

*p<0.05

**p<0.01

### Program attrition and program use

During the 11-week program, the number of login times per student varied from 2 to 24 times (mean = 8.56, SD = 5.12). Each session lasted between 25 and 50 minutes.

### Adherence

Adherence was defined as the frequency of sessions completed by students, ranging from 0 to 11. Out of 275 students, 19 students (6.9%) did not complete any sessions, 43 students (15.6%) completed 1–3 sessions, 92 students (33.5%) completed 4–7 sessions, and 121 students (44%) completed 8–11 sessions.

### Intervention effects

**Fig 3:**
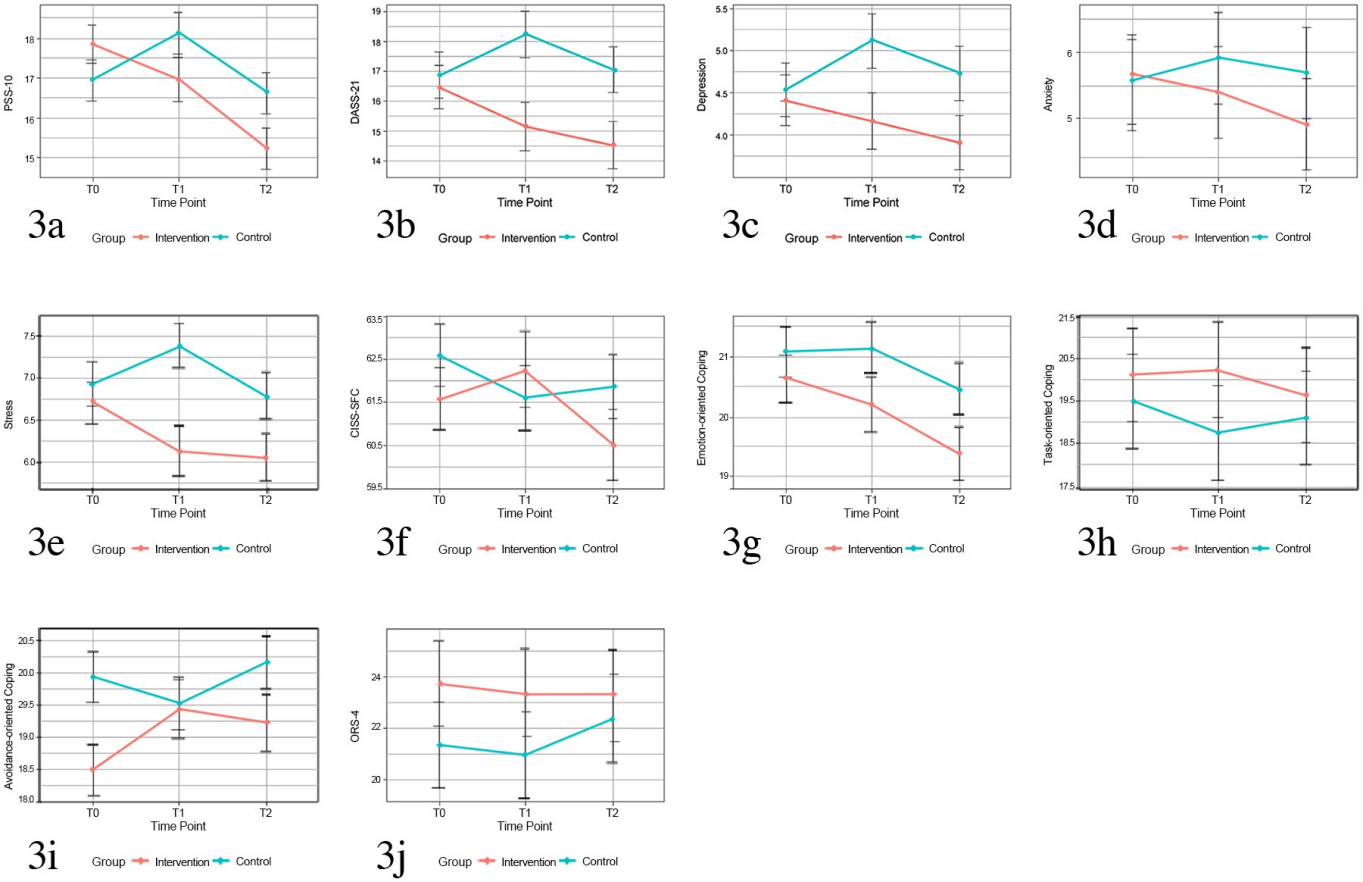
Changes of outcome variables across three timepoints.

Intent-to-treat analyses were conducted using linear mixed models (LMM) with random intercepts for students and classes to examine the effects of Coping Camp on all study outcomes. The final models for each study outcome are shown in Table 2, with results reported in the following sections, and Fig 3 summarizes the changes in all outcomes.

**Table 2:** Linear mixed model analysis results of outcome measures.

	Regression coefficient	Standard error	t-value (df)	p values
**PSS-10 (N = 499)**				
Group (control)^a^	-0.99	0.71	-1.41(13.60)	0.29
Gender (female)^d^	2.16	0.47	4.62(487.89)	0.00
Time (T1) ^b^	-0.83	0.45	-1.85(873.38)	0.12
Time (T2) ^b^	-2.62	0.41	-6.43(846.61)	0.00
Group(control)*Time (T1) ^c^	2.00	0.58	3.46(858.41)	0.01
Group(control)*Time (T2) ^c^	2.30	0.55	4.21(842.54)	0.00
AIC of initial model	AIC of final model	BIC of initial model	BIC of final model	Model comparison (p value)
8232.40	8218.10	8336.20	8270.00	0.84
**DASS-21 total score (N = 498)**				
Group (control)^a^	0.01	1.05	0.01(13.37)	0.99
Gender (female)^d^	1.85	0.77	2.41(492.25)	0.05
Time (T1) ^b^	-1.13	0.63	-1.80(854.27)	0.13
Time (T2) ^b^	-1.96	0.58	-3.41(836.90)	0.01
Group(control)*Time (T1) ^c^	2.51	0.81	3.10(841.53)	0.01
Group(control)*Time (T2) ^c^	2.16	0.77	2.82(830.29)	0.02
AIC of initial model	AIC of final model	BIC of initial model	BIC of final model	Model comparison (p value)
9197.30	9186.00	9301.00	9237.80	0.57
**DASS-21 depression subscale (N = 498)**				
Group (control)^a^	-0.08	0.44	-0.18(12.61)	0.92
Time (T1) ^b^	-0.22	0.25	-0.89(859.60)	0.50
Time (T2) ^b^	-0.55	0.23	-2.38(840.12)	0.05
Group(control)*Time (T1) ^c^	0.81	0.32	2.52(846.28)	0.04
Group(control)*Time (T2) ^c^	0.74	0.31	2.40(834.41)	0.05
AIC of initial model	AIC of final model	BIC of initial model	BIC of final model	Model comparison (p value)
6742.10	6729.20	6845.90	6775.90	0.62
**DASS-21 anxiety subscale (N = 498)**				
Group (control)^a^	-0.19	0.41	-0.47(12.53)	0.71
Gender (female)^d^	0.63	0.28	2.21(486.11)	0.07
Guardian (relatives)^e^	-1.24	1.60	-0.78(491.52)	0.53
Guardian (others) ^e^	2.26	1.11	2.03(664.23)	0.10
Time (T1) ^b^	-0.26	0.24	-1.08(854.00)	0.42
Time (T2) ^b^	-0.78	0.22	-3.60(835.44)	0.00
Group(control)*Time (T1) ^c^	0.58	0.31	1.91(841.05)	0.11
Group(control)*Time (T2) ^c^	0.89	0.29	3.08(829.15)	0.01
AIC of initial model	AIC of final model	BIC of initial model	BIC of final model	Model comparison (pvalue)
6621.70	6613.60	6725.40	6675.80	0.45
**DASS-21 stress subscale (N = 498)**				
Group (control)^a^	0.19	0.37	0.52(13.75)	0.68
Gender (female)^d^	0.72	0.27	2.64(497.05)	0.03
Time (T1) ^b^	-0.48	0.26	-1.89(876.97)	0.11
Time (T2) ^b^	-0.64	0.23	-2.74(853.42)	0.02
Group(control)*Time (T1) ^c^	0.95	0.33	2.89(861.67)	0.02
Group(control)*Time (T2) ^c^	0.51	0.31	1.63(847.51)	0.17
AIC of initial model	AIC of final model	BIC of initial model	BIC of final model	Model comparison (p value)
6719.80	6710.00	6823.50	6761.90	0.42
**CISS-SFC total score (N = 498)**				
Group (control)^a^	1.28	1.05	1.22(472.60)	0.35
Time (T1) ^b^	1.17	0.86	1.35(879.16)	0.29
Time (T2) ^b^	-0.84	0.79	-1.06(857.13)	0.42
Group(control)*Time (T1) ^c^	-2.18	1.12	-1.95(857.13)	0.10
Group(control)*Time (T2) ^c^	0.08	1.06	0.08(842.24)	0.97
AIC of initial model	AIC of final model	BIC of initial model	BIC of final model	Model comparison (p value)
9814.40	9803.90	9917.90	9850.50	0.40
**CISS-SFC emotion-oriented coping subscale (N = 499)**				
Group (control)^a^	0.50	0.56	0.89(13.64)	0.50
Gender (female)^d^	1.93	0.42	4.65(499.32)	0.00
Time (T1) ^b^	-0.26	0.39	-0.66(869.19)	0.58
Time (T2) ^b^	-1.19	0.36	-3.30(844.37)	0.01
Group(control)*Time (T1) ^c^	0.34	0.51	0.67(855.62)	0.58
Group(control)*Time (T2) ^c^	0.58	0.48	1.20(840.96)	0.35
AIC of initial model	AIC of final model	BIC of initial model	BIC of final model	Model comparison (p value)
7799.30	7787.70	7902.90	7839.50	0.59
**CISS-SFC task-oriented coping subscale (N = 498)**				
Group (control)^a^	-0.45	0.66	-0.67(14.25)	0.58
Gender (female)^d^	-1.42	0.46	-3.10(498.15)	0.01
Guardian (relatives)^e^	-5.75	2.52	-2.28(471.72)	0.06
Guardian (others) ^e^	-1.50	1.82	-0.82(639.02)	0.52
Time (T1) ^b^	0.31	0.42	0.75(866.37)	0.54
Time (T2) ^b^	-0.39	0.38	-1.02(843.39)	0.43
Group(control)*Time (T1) ^c^	-1.05	0.54	-1.95(852.50)	0.10
Group(control)*Time (T2) ^c^	0.01	0.51	0.02(838.32)	0.99
AIC of initial model	AIC of final model	BIC of initial model	BIC of final model	Model comparison (p value)
7985.40	7974.00	8089.00	8036.20	0.80
**CISS-SFC avoidance-oriented coping subscale (N = 499)**				
Group (control)^a^	1.41	0.57	2.49(781.00)	0.04
Gender (female)^d^	1.10	0.50	2.19(507.92)	0.07
Time (T1) ^b^	1.07	0.42	2.54(872.31)	0.04
Time (T2) ^b^	0.76	0.38	1.97(856.11)	0.10
Group(control)*Time (T1) ^c^	-1.48	0.54	-2.73(854.63)	0.02
Group(control)*Time (T2) ^c^	-0.52	0.51	-1.01(843.69)	0.43
AIC of initial model	AIC of final model	BIC of initial model	BIC of final model	Model comparison (p value)
8081.90	8065.60	8185.50	8117.40	0.96
**ORS-4 (N = 495)**				
Group (control)^a^	-1.84	0.95	-1.95(10.97)	0.13
Gender (female)^d^	-1.17	0.58	-2.02(484.07)	0.10
Time (T1) ^b^	-0.49	0.55	-0.90(842.17)	0.50
Time (T2) ^b^	-0.40	0.50	-0.80(819.01)	0.53
Group(control)*Time (T1) ^c^	0.10	0.70	0.14(828.32)	0.93
Group(control)*Time (T2) ^c^	1.42	0.66	2.13(812.73)	0.08
AIC of initial model	AIC of final model	BIC of initial model	BIC of final model	Model comparison (p value)
8489.10	8483.10	8571.80	8534.80	0.43

T0: Baseline, T1: 11 weeks after baseline (i.e., right after the intervention), T2: 19 weeks after baseline (i.e., follow-up)

^a^ Reference group: Intervention group

^b^ Reference group: T0

^c^ Reference group: Group (intervention) * Time (T0)

^d^ Reference group: Male

^e^ Reference group: Parents

^f^ Reference group: 15 years old

Note: This table presented the results of the final linear mixed models. Significant random effects (p<0.05) in the first linear mixed models for each outcome were chosen as random effects in the final linear mixed models. P values are two-sided and were adjusted using the Benjamini-Hochberg method to control for false discovery rate.

#### Stress (PSS-10)

The final mixed model for perceived stress, measured by PSS-10, included timepoint, group, and timepoint*group as fixed effects, along with the significant covariate gender. Results of the LMM analysis (Table 2) showed that participants in the intervention group had a highly significant reduction in stress levels compared with the control group (p = 0.01, d = 0.15) after the intervention, which was maintained at the 19-week follow-up (p < .001, d = 0.18). Fig 3a summarizes the changes in PSS-10 scores.

#### DASS-21, CISS-SFC, and ORS-4

Fig 3b–3e summarizes the changes in scores of DASS-21 and its three subscales (depression, anxiety and stress). The LMM analysis (Table 2) showed that the participants in the intervention group had significant reductions as compared with those in the control group in scores of DASS-21 (p = 0.001, d = -0.14), depression subscale (p = 0.04, d = -0.11), stress subscale (p = 0.02, d = -0.14), but not in anxiety subscale (p = 0.11, d = -0.08) after the intervention. At the 19-week follow-up, the timepoint*group interaction effects were significant for DASS-21 total scale (p = 0.02, d = -0.12) and anxiety subscale (p = 0.01, d = -0.13). However, the timepoint*group interaction effects for DASS-21 depression subscale (p = 0.05, d = 0.11) and stress subscale were not significant at 19-week follow-up (p = 0.17, d = 0.10), indicating the treatment effect did not maintain at this timepoint.

Fig 3f–3i summarize the changes in CISS-SFC scores and its three subscales: emotion-oriented coping, task-oriented coping, and avoidance-oriented coping. The LMM analysis (Table 2) showed that the timepoint*group interaction effect was only significant for CISS-SFC avoidance subscale at post-intervention assessment (p = 0.02, d = −0.11), this indicated that the intervention was effective in increasing the frequency of using avoidance coping strategies.

Fig 3j summarizes the changes in scores of ORS-4. Results of LMM analysis (Table 2) showed that there was no significant timepoint*group interaction effect (p = 0.93, d = 0.00) after the intervention or at 19-week follow-up (p = 0.08, d = 0.09). This indicates that the participants in the intervention group did not achieve a significant change in their mental health well-being as compared with those in the control group.

### Acceptability

Participants rated all 11 sessions on a 5-point scale, with average ratings ranging from 4.06 (SD = 0.89) to 4.49 (SD = 0.76), indicating high levels of perceived helpfulness. Qualitative feedback echoed these findings, with most participants reporting that Coping Camp was "great" (n = 69; 57%) and "helpful" (n = 21; 17%). However, a small number of participants reported difficulty mastering and relating to the app’s content (n = 11; 9%).

### Negative events

Out of 221 intervention group participants and 278 control group participants, 56 (25.34%) and 129 (46.40%) respectively reported negative events in response to an open-ended question. Six students (2.71%) in the intervention group and 5 students (1.80%) in the control group did not respond. The difference was not significant (P = .91). The most common negative events reported were decreased monthly academic exam scores and conflicts with teachers, parents, and friends. No serious negative events were reported in either group.

## Discussion

### Principal findings

This study tested the efficacy of Coping Camp, a stress-managing app based on stress inoculation training (SIT), among Chinese school adolescents. The primary objective was to determine whether 11 weeks of use reduced stress levels. Secondary objectives included assessing acceptability in high school settings, reducing negative mental health outcomes, improving mental health wellbeing, and changing stress coping strategies. Results showed a significant reduction in levels of stress and depression. However, Coping Camp was not effective in improving mental health wellbeing or changing stress coping strategies.

### Perceived stress

To our knowledge, this study was one of the first to demonstrate the stress-reducing effects of a mobile app based on stress inoculation training (SIT) in a universal sample of Chinese school adolescents. The results showed that the use of the Coping Camp significantly reduced the perceived stress level both at post-intervention and 19-week follow-up. The effect sizes were Cohen’s d = 0.15 at post-intervention and 0.18 at 19-week follow-up. The effect sizes were small as compared with previous online interventions among adolescents and young adults [42]. First, this intervention was arranged during moral classes which were followed by evening classes where students were engaged with homework and preparation for the next day’s classes, it is possible that students were not fully engaged with the intervention. Second, we recruited universal sample instead of a clinical sample, previous research has shown that interventions that recruited students with elevated symptoms are likely to produce larger effect sizes than those that recruited a universal sample [43]. Last, this is the exploratory study to investigate an app that was designed for Chinese high school students, further improvement needs to be made according to the user experience of participating students in order for the Coping Camp to generate larger effect size.

### Depression and anxiety

Our study found that Coping Camp significantly reduced DASS-21 score at both post-intervention (*P* < .001) and 19-week follow-up (*P* < .01). Compared with the students in the controlled group, those who used Coping Camp decreased 2.69 at post intervention, which is similar to a previous study which tested the efficacy of online intervention among Chinese school adolescents [19]. Also, the effect sizes were Cohen’s d ranged from 0.08 to 0.13. The effect sizes were small compared with previous online interventions [19]. Similar reasons provided for perceived stress may provide an explanation.

### Mental health wellbeing (ORS-4)

We found that Coping Camp was not effective in improving mental health well-being. This finding is similar to previous similar studies [44–46]. Outcome rating scale (ORS-4) was developed to measure the changes of global psychological distress and wellbeing during the psychotherapy sessions [34]. The possible reason why current intervention did not reduce the score of ORS-4 may be because current intervention was only designed for reducing stress and the scenarios might not suit to all the problems that these students confronted, according to the comments from students. Therefore, in order to achieve a more comprehensive change of students’ psychological wellbeing, it is recommended that stakeholders (e.g., teachers, parents etc.) could be involved and the scenarios should be more diverse.

### Stress coping behaviours (CISS-SFC)

At the time of writing, there is limited number of studies examining the evidence on online and app-based mental health interventions’ effects on coping behaviour changes. Our study is one of the first to provide evidence on this subject and found that the use of an app-based mental health intervention, the Coping Camp, was not effective in changing stress coping strategies. This finding is in line with several studies that suggested face-to-face interventions were not effective in changing stress coping strategies among adolescents [47–49]. Several reasons were likely to contribute to the inefficacies. First, except for cognitive skills which took two sessions, other skills (e.g., mindfulness skills, progressive muscle relaxation skills, time management skills, problem-solving skills) are taught within one session which lasted for 30–40 minutes; and 30–40 minutes might not be enough for students to fully master each skill. In addition, except for the use during the intervention sessions, mobile phones were not available to participants during weekdays; the participants, therefore, did not have enough chance to practice and master these skills and apply these skills to their daily life. Second, the self-help nature of the intervention might be difficult for students too. Most of the students had no experience with coping skills training prior to the intervention. Future trials are warranted to investigate mobile app-based interventions’ effects on coping behaviour changes, which are critical for adolescents to maintain mental health wellbeing amid adverse life events [50]. For mental health apps providing coping strategy trainings, more guidance should be involved in the intervention to generate a larger effect. Third, according to behaviourism, reinforcements from environments are needed for new behaviours to sustain and consolidate, therefore, gamification features, such as incentives and rewards, may be involved in such apps to reinforce newly learnt coping behaviours. Also involving teachers/parents and creating a supportive classroom environment that encourages students to use positive coping behaviours would be beneficial.

Notably, we found that gender had significant effects on stress and coping behaviours. First, compared with male, females were under significantly more stress. Second, gender had significant effects on the coping styles. Compared with males, female participants used significantly more emotion- and avoidance-oriented coping strategies. Also, female participants used less task-oriented coping strategies. These findings are in line with previous research [51, 52]. We suggest future studies investigate gender-associated preferences in intervention development and investigate ways to improve female adolescents’ functional coping behaviours.

### Acceptability

The results of this study showed that the Coping Camp was acceptable to Chinese high school students and the use of Coping Camp was feasible in Chinese high school settings. The rates of the Coping Camp were ranged from 4.05 to 4.49 across sessions, which was above the cut-off score of “useful”. This rating was similar to previous online interventions that was conducted in school settings [53]. Qualitative feedback reflected that the participants liked the app as they learnt skills and the app was helpful in reducing stress in their lives. This indicates that mental health apps need to be helpful in relieving stress to be acceptable.

### Strength, limitations and implications for policymakers

To our best knowledge, this study is the first randomized controlled trial to investigate the efficacies of an app, the Coping Camp, which was developed for Chinese high school students and reflects the real-world effect of mobile intervention. This study showed that the use of the Coping Camp significantly reduced the stress levels, anxiety and depression among Chinese high school students and was feasible and acceptable among Chinese high school students. This has important implications for policymakers and educators. They can make use of internet resources (e.g., webpages and apps) as a mode to deliver psychoeducation and training about stress and coping where there is a lack of human resources in mental health professions. Several limitations should be noted. First, the sample was recruited from a moderate city in China, the results should be generated to big cities and remote/rural areas with caution. Second, as this is the first app that was developed for Chinese high school students, improvements should be made before it can be used for larger population. Third, participants were allocated randomly to two groups within the same school, which allowed for information sharing and cell phone exchanges. This might introduce contamination bias.

## Conclusions

This study showed that the use of an app-based intervention, the Coping Camp, which was developed for managing stress among Chinese high school students was feasible, acceptable and effective, which provides an alternative for Chinese high schools where there are lack of mental health professionals and students experience excessive stress.

## Supporting information

S1 File Protocol. (DOCX)

S2 File Tianjin normal University scientific research ethics review form. (DOCX)

S3 File Human research ethics approval. (PDF)

S1 Fig (TIFF)

S1 Checklist CONSORT 2010 checklist of information to include when reporting a randomised trial*. (DOC)
