# Supplementary material for: The feasibility, acceptability and efficacy of an app-based intervention (the Coping Camp) in reducing stress among Chinese school adolescents: A cluster randomised controlled trial
Source: PLoS One. 2023 Nov 27;18(11):e0294119. doi: 10.1371/journal.pone.0294119 (PMC10681230; doi:10.1371/journal.pone.0294119)
Supplement: S1 File — (DOCX) [file pone.0294119.s001.docx]

**PROTOCOL**

**The feasibility, acceptability and efficacy of an online self-help intervention for stress management among adolescents in school settings in China**

Protocol Version: SPIRIT 2013 Statement

Date:2/04/2021

Authors:

Ms Xiaoyun Zhou

Dr Matthew Bambling

Dr Sisira Edirippulige

Prof Xuejun Bai

Centre for Online Health

The University of Queensland

Princess Alexandra Hospital, Building 33

Woolloongabba, QLD 4102

# Table of Contents

[Administrative Information 3](#_Toc68208833)

[Trial registration 3](#_Toc68208834)

[Funding 3](#_Toc68208835)

[Roles and responsibilities 3](#_Toc68208836)

[Names, affiliations, and roles of protocol contributors 3](#_Toc68208837)

[Name and contact information for the trial sponsor 3](#_Toc68208838)

[Role of study sponsor and funders 4](#_Toc68208839)

[Individual and/or groups overseeing the trial 4](#_Toc68208840)

[Introduction 4](#_Toc68208841)

[Background and rationale 4](#_Toc68208842)

[Comparators 5](#_Toc68208843)

[Objectives 5](#_Toc68208844)

[Trial design 5](#_Toc68208845)

[Methods 6](#_Toc68208846)

[Participants, interventions, and outcomes 6](#_Toc68208847)

[Study setting 6](#_Toc68208848)

[Eligibility criteria 6](#_Toc68208849)

[Interventions 6](#_Toc68208850)

[Outcomes 7](#_Toc68208851)

[Participant timeline 9](#_Toc68208852)

[Sample size 10](#_Toc68208853)

[Recruitment 11](#_Toc68208854)

[Assignment of interventions 11](#_Toc68208855)

[Allocation 11](#_Toc68208856)

[Implementation 11](#_Toc68208857)

[Blinding 11](#_Toc68208858)

[Methods: Data collection, management and analysis 11](#_Toc68208859)

[Data collection methods 11](#_Toc68208860)

[Data management 13](#_Toc68208861)

[Statistical methods 13](#_Toc68208862)

[Monitoring 14](#_Toc68208863)

[Data monitoring 14](#_Toc68208864)

[Harms 14](#_Toc68208865)

[Auditing 14](#_Toc68208866)

[Ethics and Dissemination 15](#_Toc68208867)

[Research ethics approval 15](#_Toc68208868)

[Protocol amendments 15](#_Toc68208869)

[Consent or assent 15](#_Toc68208870)

[Confidentiality 15](#_Toc68208871)

[Declaration of interests 15](#_Toc68208872)

[Access to data 15](#_Toc68208873)

[Ancillary and post-trial care 16](#_Toc68208874)

[Dissemination policy 16](#_Toc68208875)

[References 17](#_Toc68208876)

[Appendices 20](#_Toc68208877)

[Appendix 1 20](#_Toc68208878)

[Appendix 2 24](#_Toc68208879)

# Administrative Information

## Trial registration

Trial identifier: Request Id: 380316 (submitted to Australian New Zealand Clinical Trials Registry; ANZCTR, not registered yet)

Registry name: web-based stress management intervention for Chinese adolescents in school settings: A randomized controlled trial investigating effect on perceived stress levels

## Funding

There is no funding for this trial.

## Roles and responsibilities

### Names, affiliations, and roles of protocol contributors

| **Name** | **Affiliation** | **Role** |
| --- | --- | --- |
| Ms Xiaoyun Zhou | Centre for Online Health, Faculty of Medicine, The University of Queensland, Australia | Principal investigator, PhD student |
| Dr Matthew Bambling | Centre for Online Health, Faculty of Medicine, The University of Queensland, Australia | Principal investigator, Principal advisor |
| Dr Sisira Edirippulige | Centre for Online Health, Faculty of Medicine, The University of Queensland, Australia | Investigator, associate advisor |
| Prof Xuejun Bai | Academy of Psychology and Behaviour, Tianjin Normal University, China | Investigator, associate advisor |

### Name and contact information for the trial sponsor

*Name of the trial sponsor:*

The University of Queensland, Australia

*Contact information:*

Address: 280-284 Sir Fred Schonell Dr, St Lucia QLD 4067, Australia

Tel: +61 [(07) 3365 1111](tel:+61733651111)

### Role of study sponsor and funders

The data collected in this study will be stored and managed using the UQ Research Data Manager (UQRDM), which is an online data storage system owned by The University of Queensland, Australia.

### Individual and/or groups overseeing the trial

| **Responsible individuals/groups** | **Responsibilities** |
| --- | --- |
| Centre for Online Health, Faculty of Medicine, The University of Queensland | Coordinating centre |
| Dr Matthew Bambling, Dr Sisira Edirippulige and Prof Xuejun Bai | Supervising the conduct of the intervention |

# Introduction

## Background and rationale

Adolescence is a transitional period with rapid physical and psychosocial changes, which is a developmental period sensitive to stress (1). It is worth noting that levels of stress seem to increase from preadolescence to adolescence (2) and combine with inadequate coping, may induce stress’s well-known effects (3-5). Stress and mental health problems are strongly and significantly correlated for school students (6). Previous studies suggested that stress forms a non-specific risk factor for a wide range of mental health issues, behavioural problems and conduct disorders among adolescents (7-15). Adolescents who are stressed are at higher risk of a wide range of mental health conditions such as anxiety, depression (16), bipolar disorder (16), schizophrenia (16), eating disorders (17), personality disorders (16) and even suicide (18). Those mental health conditions were cited by adolescents as the second-most cited barriers to achieving study goals (19). High school students who experience negative affects and engage maladaptive coping are more likely have lower GPA (20). School related academic stress and burnout decreases student academic motivation and increases the risk for dropout (21, 22). Among adolescents, stress level is also associated with risky behaviours. Moderate- and high-level stress is associated with gaming addition among adolescents (23), and affects their family and social relationships, self-control and self-regulation, as well as academic performance (24, 25).

Managing stress among adolescents is considered to be critical to preventing mental health issues.

Previous studies have shown the benefits of delivering psychological interventions (26) in group formats for stress management among adolescents. In addition, developing countries, such as China, are lack of mental health professionals, especially school counsellors. Therefore, telehealth interventions (e.g., video conferencing, mobile apps etc.) might provide an option that reduce the demand of mental health professionals. In fact, telehealth interventions have been used for the management of adolescent mental health conditions such as anxiety, depression and stress previously. However, most of such interventions were conducted in western countries. Although two studies (27, 28) have transferred web-based self-help interventions (CATCH-IT and MoodGYM) to Chinese version and showed effectiveness among Chinese young people. However, the number is still very small (i.e., only 2 studies); besides, only Ip et al (27) conducted intervention in secondary school students with relatively large sample size. Ren et al (28) only tested the intervention with a sample of 62 among university student population. Little is known about the effectiveness of web-based interventions in Chinese adolescent context.

### Comparators

The comparator will be no treatment condition. This means that the control group will not be given the intervention product (a web-based stress managing website or an app).

## Objectives

This study aims to test whether an online school-based intervention for stress management is feasible, acceptable and effective among Chinese high school students. We hypothesise that our intervention (compared to the control group) will result in lower stress.

## Trial design

A superiority, two-arm, cluster randomized controlled trial will be conducted (see Figure 1 for the flow chart of the trial design). Confirmed participants will be randomly allocated in a 1:1 ratio into one of the study groups. In the intervention group, the participants will use an online stress-managing program which will be delivered via smartphone. Participants in the control group will not use the online program duration. However, they will be notified that they will have access to the online program after 12 weeks (i.e., at the end of the study). The condition to which each participant will be allocated will not be blinded for either experimenters or participants.


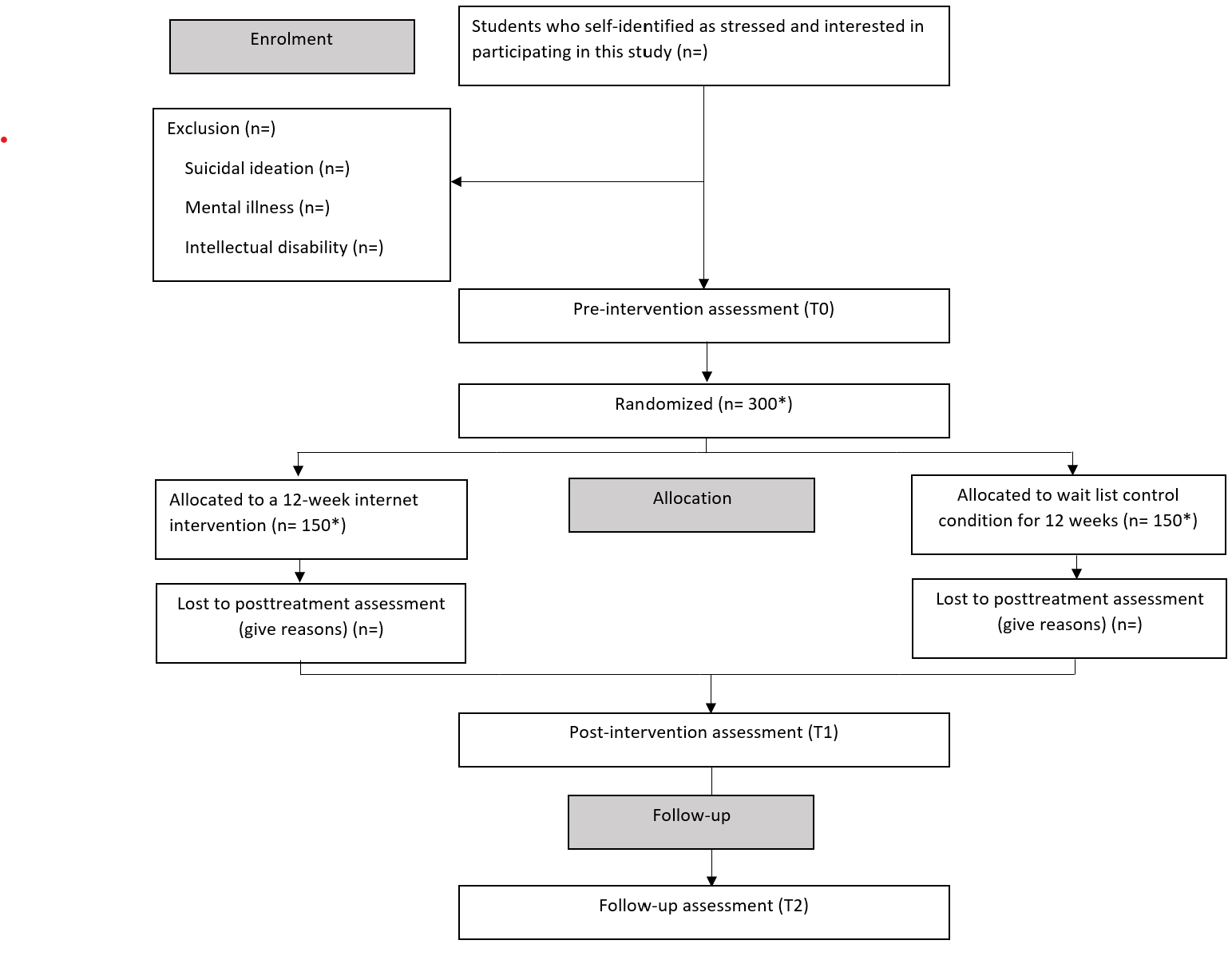


Figure 1 Flow chat of the trial design

# Methods

## Participants, interventions, and outcomes

### Study setting

This study will be conducted in two high schools: Mianyang Dongchen International School (MDIS) and Mianyang Nanshan High School (MNHS) in Mianyang City, Sichuan Province in China. MDIS is a private school and MHS is a public school.

### Eligibility criteria

Eligible participants include those (i) who will be enrolled in grade 10 and 11 at the beginning of the intervention; (ii) who own a smartphone and are allowed to use the smartphone during intervention period; Participants will be excluded if they (i) are intellectually impaired; (ii) are diagnosed with mental illness; (iii) are having medications which affect their mental health status (e.g., antidepressant) or attend regular counselling sessions.

### Interventions

The intervention group will download a stress-managing app, the app can be used on mobile devices including mobile phones and tablets etc. The app will be based on Stress Inoculation Training (SIT) developed by Meichenbaum (29). Even though SIT was designed for military settings, there has been a lot of studies which adapted the SIT and use it among students and adolescents (30-34) and delivered SIT via technologies (35). Our study will modify SIT to high school context by adding assertiveness and training participants cognitive and behavioural techniques to deal with stressors arising from academic environment and peer interpersonal relationships. The online stress-managing intervention will comprise of 11 sessions which consist of psychoeducation, problem assessments, training of stress coping skills (emotion-focused coping skills and problem-focused coping skills), cognitive restructuring, imagery and behavioural rehearsal and role play (see [Appendix 1](#_Appendix_1) for details of the intervention content). The intervention content will be in Chinese language. The control group will not be given access to the app until the termination of the trial (12 weeks after the start of the trial). There will be no other intervention given to the control group. Both the intervention group and the control group will have full access to usual care, which is the psychological courses arranged by the high schools.

The criteria for discontinuing for a given trial participant include: (i) if they self-reported or reported by others to be suicidal; (ii) if they drop out of school during the intervention period; (iii) if they require to withdraw from the study.

Strategies to improve adherence to intervention protocol include:

- Instructions about the importance of following study guidelines for adherence to once weekly product.
- Instructions about the purpose and use of the intervention app.
- Notification sent via app weekly on each Sunday that there will be a session taken in the app.
- Successfully completion of each session will generate a virtual star in the participant’s account as a reward for the participation.
- Adherence assessments include log in times and other usage data collected in the app.

Relevant concomitant care and interventions that are permitted or prohibited during the trial are as following: Both the intervention group and the control group will have full access to psychological courses as arranged according to the curriculums of the high schools. However, participants in either intervention group or control group will be prohibited to use psychological counselling services. Should they have any mental health conditions which require mental health assistance, they will be referred to relevant services AND the participants will be regarded as drop-outs.

### Outcomes

*Primary Outcome Measure*

The success of the intervention is defined as statistically significant more reduction in mean score of PSS-10 in the intervention group as compared with the control group at 12 weeks. The primary outcome measure will be Perceived Stress Scale-10 Items (PSS-10). The PSS-10 is a ten item self-reported scale to assess respondents’ global level of perceived stress (36). Respondents were required to rate the frequency of certain feelings they experienced over the past month on a 5-point Likert Scale (1=never, 2=rarely, 3=sometimes, 4=often, 5=always). It has shown to predict the range of health-related outcomes presumed to be associated with appraised stress (36). The PSS-10 has been translated in Chinese and shown acceptable reliability and validity in both adults (37, 38) and adolescents (39).

*Secondary Outcome Measures*

For secondary outcome meansures, the success of the intervention is defined as statistically significant more reduction in mean score of DASS-21 and statistically significant more increase in mean score of ORS and CISS-SFC in the intervention group as compared with the control group at 12 weeks. Negative mental health outcome will be measured using Depression, Anxiety and Stress Scale (DASS-21). DASS-21 consists of 21 items. Each item is rated on a 4 Likert scale ranging from 0 to 3, indicating to what degree each statement applied to participants over the past week. Summed scores range from 0-42 with more severe symptoms indicated by higher scores. This study will utilize total scores in addition to subscale scores. DASS-21 has been validated in Chinese adolescents and proved to have satisfactory psychometric properties in Chinese adolescent population (40).

Positive mental health status will be measured using Outcome rating scale (ORS). The ORS is a four-item self-report instrument. The ORS was developed as a brief alternative to the Outcome Questionnaire-45 (OQ-45)-a widely used and well-validated measure (41). The ORS was designed to assess change in three areas of client functioning generally considered valid indicators of treatment progress: individual (or symptomatic) functioning, interpersonal relationships, and social role performance (work adjustment, quality of life). The ORS was found to have adequate reliability and validity (42). The Chinese version of the ORS was recently validated in Chinese sample and showed to be a reliable and valid measure for Chinese sample and sub-clinical clients (43).

Stress coping behaviors will be measured using the short form of Coping Inventory for Stressful Situations (CISS-SFC). The CISS-SFC is a 21-item scale which measures three dimensions of coping-task-oriented coping, emotion-oriented coping, and avoidance-oriented coping. CISS-SFC has been translated into Chinese and approved to be satisfactorily valid and reliable among Chinese university students (44).

### Participant timeline

Table 1 and Figure 2 illustrate the time schedule of enrolment, randomization, interventions, and assessments for participants.

Table 1 Perceived Stress Reduction Protocol Schedule of Forms and Procedures

|  | | | **-1** | **T0** | **T1** | **T2** |
| --- | --- | --- | --- | --- | --- | --- |
| **Activity/**  **Assessment** | **Staff**  **Member** | **Approximate**  **Time to**  **Complete** | **Pre-Study**  **Screening/ Consent** | **Pre-Study**  **Baseline/**  **Randomization** | **Post-study**  **12 weeks** | **Follow-Up**  **16 weeks** |
| Consent Form | Head teachers | 20 Minutes | X |  |  |  |
| Inclusion/  Exclusion Form | Principal investigator | 5 minutes | X |  |  |  |
| Demographics Questionnaire | Principal investigator | 10 Minutes |  | X | X | X |
| PSS-10 | Principal investigator | 10 minutes |  | X | X | X |
| DASS-21 | Principal investigator | 20 minutes |  | X | X | X |
| ORS-4 | Principal investigator | 5 minutes |  | X | X | X |
| CISS-SFC | Principal investigator | 20 minutes |  | X | X | X |
| Randomization | Principal investigator | 15 Minutes |  | X |  |  |


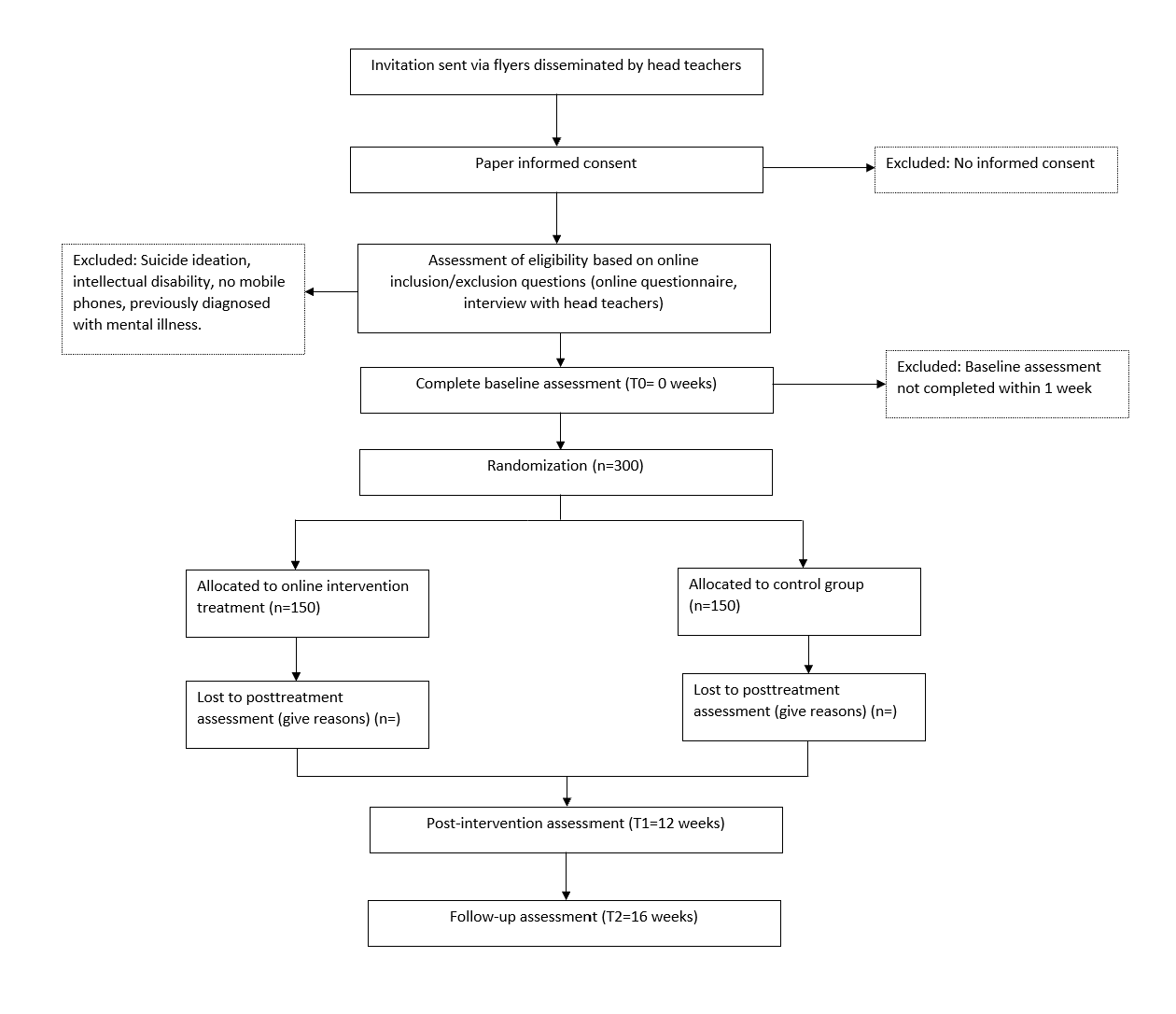


Figure 2 Participant Timeline

### Sample size

According to a meta-analysis on internet interventions on stress reduction (45), which reported that the overall mean effect size for stress at posttest was Cohen d=0.43 (95% CI 0.31-0.54). Significant, small effects were found for depression (Cohen d=0.34, 95% CI 0.21-0.48) and anxiety (Cohen d=0.32, 95% CI 0.17-0.47). Following this meta-analysis, we therefore choose effect sizes of d=0.43 for perceived stress, d=0.34 for depression and d=0.32 for anxiety. An absolutely reduction of 4.03 points is chosen as minimum clinically significant difference according to a previous study that analyzed PSS-10 scores in terms of minimum clinically significant important difference (MCID) (46). Based on this expected difference, no expected PSS-10 change in the control group, a population mean PSS-10 of 13.7 (SD=5.6) equal between groups (47), a sample size of 172 (n=86 in each study group) is required to detect effect size of d=0.43 with a power (1− β) of 0.80 with alpha of 0.05. A sample size of 192 (n=96 in each study group) will be needed when allowing for 10% drop out. Considering the availability of target sample, we will choose a sample size of 300 (n=150 for each study group).

### Recruitment

Participants will be recruited from grade 10 and 11 at Mianyang Dongchen International School (MDIS) and Mianyang Nanshan High School (MNHS). MDIS has a student population of more than 2000 in grade 10 and 11. MHS has a student population of more than 3500 in grade 10 and 11. More than 90 percent of high school students (total n= 4950 students) in MDIS and MHS own a mobile phone, and both schools allow students to use mobile phones on the weekends. Recruitment strategies for the trial will involve posters, flyers, advertisement and announcements in class in those two schools. Students who are self-identified as feeling stressed will be recruited. For all participants, consent forms will be read and signed by themselves, their guardians and their head teachers.

## Assignment of interventions

### Allocation

A two-arm randomized controlled trial will be conducted. Confirmed participants will be randomly allocated in a 1:1 ratio into one of the study groups. Students will be randomised within schools. In order to minimise contamination, students will be randomised on a class basis. Also, students will be notified that all of the participantas will have access to the online program, therefore they should not talk to each other about the content of the intervention. In the intervention group, the participants will use an online stress-managing program which will be delivered via smartphone. Participants in the control group will not use the online program duration. However, they will be notified that they will have access to the online program after 16 weeks (i.e., at the end of the study). The condition to which each participant will be allocated will not be blinded for either experimenters or participants.

### Implementation

Clusters of students will be randomly assigned (1:1) to either the intervention of control condiction by use of computer-generated randomisation list prepared by a research assistant independent from the research groups. The participants will be enrolled by principal investigator (XZ), with the assistance of head teachers.

### Blinding

Nether the participants nor the researchers will be blinded after assignment to the intervention.

## Methods: Data collection, management and analysis

### Data collection methods

Data relating to perceived stress, negative mental health outcome, positive mental health outcome and coping behaviours will be collected at baseline (T0=0 weeks), post-intervention (T1=12 weeks) and follow-up (T2=16 weeks). Demographic data will only be collected at baseline (see Table1 for study variables). All data will be collected online via the mobile intervention app.

Baseline variables, including demographics, will be gather at the start of the study (Table 1).

Table 2 Study variables

| **Variable** | **Measure** |
| --- | --- |
| Demographic | Age |
|  | Gender |
|  | Grade |
|  | Home location (rural or urban) |
|  | Guardian (parents or others) |
| Perceived stress | PSS-10 |
| Negative mental health | DASS-21 |
| Positive mental health | ORS-4 |
| Coping Behaviors | CISS-SFC |

A description of study instruments along with their reliability and validity are provided below:

The Perceived Stress Scale-10 Items (PSS-10) is a ten item self-reported scale to assess respondents’ global level of perceived stress (36). Respondents were required to rate the frequency of certain feelings they experienced over the past month on a 5-point Likert Scale (1=never, 2=rarely, 3=sometimes, 4=often, 5=always). It has shown to predict the range of health-related outcomes presumed to be associated with appraised stress (36). PSS-10 is a shorter version of Perceived Stress Scale 14-item (PSS-14) that assesses how respondents perceives their lives to be unpredictable, uncontrollable and overloaded (48). The PSS-10 has been validated in populations in different countries and proven to possesses adequate internal consistency with Cronbach’s alpha coefficients ranging from 0.67 to 0.91 (49-54), moderate convergent validity with stressful life events (55), and good concurrent validity with mental health problems such as anxiety and depression (56-58). Particularly, the PSS-10 has been translated in Chinese and shown acceptable reliability and validity in both adults (37, 38) and adolescents (39).

Negative mental health outcome (i.e., anxiety, depression and stress) will be measured using Depression, Anxiety and Stress Scale (DASS-21). DASS-21 consists of 21 items. Each item is rated on a 4 Likert scale ranging from 0 to 3, indicating to what degree each statement applied to participants over the past week. Summed scores range from 0-42 with more severe symptoms indicated by higher scores. This study will utilize total scores in addition to subscale scores. DASS-21 has been validated in Chinese adolescents and proved to have satisfactory psychometric properties in Chinese adolescent population (40).

Positive mental health status will be measured using Outcome rating scale (ORS). The ORS is a four-item self-report instrument. The ORS was developed as a brief alternative to the Outcome Questionnaire-45 (OQ-45)-a widely used and well-validated measure (41). The ORS was designed to assess change in three areas of client functioning generally considered valid indicators of treatment progress: individual (or symptomatic) functioning, interpersonal relationships, and social role performance (work adjustment, quality of life). The ORS was found to have adequate reliability and validity (42). The Chinese version of the ORS was recently validated in Chinese sample and showed to be a reliable and valid measure for Chinese sample and sub-clinical clients (43).

Stress coping behaviours will be measured using the short form of Coping Inventory for Stressful Situations (CISS-SFC). The CISS-SFC is a 21-item scale which measures three dimensions of coping-task-oriented coping, emotion-oriented coping, and avoidance-oriented coping. CISS-SFC has been translated into Chinese and approved to be satisfactorily valid and reliable among Chinese university students (44).

The usability and acceptance of the program will be assessed in order to gain information about users’ needs and specificities to improve interfaces of online programs for Chinese adolescents. Acceptance of the online program will be assessed by providing the users’ opportunity to write feedback at the end of each sessions. The contents of the written feedback of the online program will be evaluated. Usability will be assessed using measures such as the time they spent with the program and by the number of sessions they completed.

### Data management

In the intervention, all data will be entered electronically. This will be done at the participating site where the principal researcher (XZ) will be located during the intervention period. To ensure the security of the data, originally data will be re-entered to Microsoft Word documents and stored on the UQRDM, the cloud data management system owned by the sponsor of this trial (The University of Queensland).

### Statistical methods

Statistical analyses will be performed using SPSS 22.0 (SPSS Inc., Chicago, IL). We will present descriptive data in a table: categorical variables (e.g., gender, grade, home location etc.) will be presented as counts and percentages. Continuous variables (e.g., age, PSS-10, DASS-21, ORS-4 etc.) will be presented as mean and standard deviation (SD).

To determine whether any significant differences exist between intervention group and control group at baseline, independent t test will be conducted on continuous baseline variables (e.g., age, PSS-10, DASS-21, ORS-4, CISS-SFC, MER), and chi-square analyses will be conducted on categorical or nominal variables (e.g., gender, home location, guardian etc.).

To determine whether the intervention is effective, intention-to-treat (ITT) analyses will be conducted separately (59). The ITT analysis will include the data from the whole randomized sample. A linear mixed model (LMM) will be used to investigate the effects of time and groups on the primary outcome variables. The LMM will be performed with group, time, and group × time as fixed effects. Subjects and schools will be selected as random effects in order to take into consideration both within-subject and within-school correlations. Cohen d effect sizes will be calculated to examine the magnitude of between-group differences. Similar analyses will be conducted for exploring effects on secondary outcome measures.

## Monitoring

### Data monitoring

There will not be interim analyses, analyses will only be done at 16 weeks after the randomization.

### Harms

In our study an adverse event will be defined as serious psychological distress experienced by participants without regard to the possibility of a causal relationship. Adverse events will be collected after the subject has provided consent and enrolled in the study. If a subject experiences an adverse event after the informed consent document is signed but the subject has not started to receive study intervention, the event will be reported as not related to study intervention. All adverse events occurring after entry into the study will be recorded. If an adverse event meets the criteria for a serious adverse event after study enrolment, this will be reported to Centre for Online Health, The University of Queensland. If the participant drop out as a result of an adverse event, study personnel will document the circumstances and data leading to discontinuation of the intervention. A serious adverse event for this study is any occurrence that is believed by the investigators to be causally related to the intervention and results in any of the following: Life-threatening condition (i.e., suicidal ideation or attempts), hospitalization due to mental health conditions, drop out of school.

### Auditing

There will not be auditing trial conduct.

# Ethics and Dissemination

### Research ethics approval

As this study will be conducted at multiple sites in two countries: Australia and China. We will therefore obtain ethics approval from Tianjin Normal University Ethics Committee in China and The University of Queensland Human Ethics Office in Australia. Participants under 18 years will give consent signed by their parents or guardians. Participants >=18 years will give consent signed by themselves. In addition, Participants will be given a Participants Information Sheet which describes the detail of the project and potential benefits and risks before giving consent to participate in this study.

### Protocol amendments

Amendments of the protocol will be discussed within research team members. Approval of amendments will also be obtained from Tianjin Normal University Ethics Committee in China and The University of Queensland Human Ethics Office in Australia.

### Consent or assent

Principal investigator (XZ) will send and collect paper consent forms from participants for assignment from their guardians, head teachers and themselves.

### Confidentiality

All information collected from this study will be treated as strictly confidential. All the data will be kept in a locked filing cabinet or on a password-protected computer in a secure office at the Centre for Online at the University of Queensland. All reporting about the research will be aggregated so that no individual can be identified. The information about the participants will be kept separately from the data. Only the research team members can access the identified data. The data used for published reports will not have any information relating to personal details of the participants.

### Declaration of interests

There is not competing interests in this study.

### Access to data

Only the research team members will have access to the final trial dataset. There is no contractual agreements that limit such access for investigators.

### Ancillary and post-trial care

Participants who suffer from psychological distress as a result of the intervention will receive free counselling from the principal investigator (XZ). The principal investigator is a registered psychological counsellor in China, XZ is also experienced with conducting individual and group psychotherapy with adolescents and school students.

### Dissemination policy

If participants are interested in the result of the study, they will send their email address to the contact person of the study (Xiaoyun Zhou). The results of this study will be published in a peer-reviewed journal. The results will be send to interested individual participants via the email address they provided. We will not use professional writers. There is no plan for granting public access to the full protocol, participant-level dataset nor statistical code.

Informed consent materials can be found in [Appendix 2](#_Appendix_2).

# References

1. Fuhrmann D, Knoll LJ, Blakemore S-JJTics. Adolescence as a sensitive period of brain development. 2015;19(10):558-66.

2. Rudolph KDJJoah. Gender differences in emotional responses to interpersonal stress during adolescence. 2002;30(4):3-13.

3. Chrousos GPJNre. Stress and disorders of the stress system. 2009;5(7):374.

4. McLaughlin KA, Hatzenbuehler MLJJoAH. Mechanisms linking stressful life events and mental health problems in a prospective, community-based sample of adolescents. 2009;44(2):153-60.

5. Seiffge‐Krenke I, Aunola K, Nurmi JEJCd. Changes in stress perception and coping during adolescence: The role of situational and personal factors. 2009;80(1):259-79.

6. Al-Gelban KS. Depression, anxiety and stress among Saudi adolescent school boys. The journal of the Royal Society for the Promotion of Health. 2007;127(1):33-7.

7. Bandell‐Hoekstra I, Abu‐Saad HH, Passchier J, Knipschild PJHtjoh, pain f. Recurrent headache, coping, and quality of life in children: a review. 2000;40(5):357-70.

8. Compas BE, Connor-Smith JK, Saltzman H, Thomsen AH, Wadsworth MEJPb. Coping with stress during childhood and adolescence: problems, progress, and potential in theory and research. 2001;127(1):87.

9. Compas BE, Orosan PG, Grant KEJJoa. Adolescent stress and coping: Implications for psychopathology during adolescence. 1993;16(3):331.

10. Kristjánsdóttir G. Recurrent Pains: A Public Health Concern in School-age Children: an Investigation of Headache, Stomach Pain, and Back Pain: Nordic School of Public Health [Nordiska hälsovårdshögsk.]; 1996.

11. Lovibond PF, Lovibond SHJBr, therapy. The structure of negative emotional states: Comparison of the Depression Anxiety Stress Scales (DASS) with the Beck Depression and Anxiety Inventories. 1995;33(3):335-43.

12. Macleod J, Oakes R, Copello A, Crome I, Egger M, Hickman M, et al. Psychological and social sequelae of cannabis and other illicit drug use by young people: a systematic review of longitudinal, general population studies. 2004;363(9421):1579-88.

13. McNamara S. Stress in young people: What's new and what to do: A&C Black; 2000.

14. Passchier J, Orlebeke J. Headache and stress in schoolchildren: An epidemiology study. Cephalaglia; 1985.

15. Van Praag HM, de Kloet ER, Van Os J. Stress, the brain and depression: Cambridge University Press; 2004.

16. Harkness KL, Hayden EP. The Oxford Handbook of Stress and Mental Health: Oxford University Press, USA; 2020.

17. Rojo L, Conesa L, Bermudez O, Livianos LJPm. Influence of stress in the onset of eating disorders: data from a two-stage epidemiologic controlled study. 2006;68(4):628-35.

18. Stewart JG, Shields GS, Esposito EC, Cosby EA, Allen NB, Slavich GM, et al. Life stress and suicide in adolescents. 2019;47(10):1707-22.

19. Carlisle E., Fildes J, Hall S, Perrens B, Perdriau A, Plummer J. Youth Survey Report 2019: Sydney, NSW: Mission Australia; 2019. Available from: file:///C:/Users/61435/Downloads/Mission%20Australia%20Youth%20Survey%20Report%202017.pdf.

20. Arsenio WF, Loria SJTJoGP. Coping with negative emotions: Connections with adolescents’ academic performance and stress. 2014;175(1):76-90.

21. Liu YJL, Differences I. The longitudinal relationship between Chinese high school students' academic stress and academic motivation. 2015;38:123-6.

22. Walburg VJC, Review YS. Burnout among high school students: A literature review. 2014;42:28-33.

23. Rajab AM, Zaghloul MS, Enabi S, Rajab TM, Al-Khani AM, Basalah A, et al. Gaming addiction and perceived stress among Saudi adolescents. 2020:100261.

24. Canale N, Marino C, Griffiths MD, Scacchi L, Monaci MG, Vieno AJJoBA. The association between problematic online gaming and perceived stress: The moderating effect of psychological resilience. 2019;8(1):174-80.

25. Gentile DJPs. Pathological video-game use among youth ages 8 to 18: A national study. 2009;20(5):594-602.

26. Feiss R, Dolinger SB, Merritt M, Reiche E, Martin K, Yanes JA, et al. A systematic review and meta-analysis of school-based stress, anxiety, and depression prevention programs for adolescents. Journal of youth and adolescence. 2019;48(9):1668-85.

27. Ip P, Chim D, Chan KL, Li TM, Ho FK, Van Voorhees BW, et al. Effectiveness of a culturally attuned Internet-based depression prevention program for Chinese adolescents: A randomized controlled trial. Depression and anxiety. 2016;33(12):1123-31.

28. Effectiveness and mechanism of internet-based self-help intervention for depression: The Chinese version of MoodGYM [press release]. China: Science Press2016.

29. Meichenbaum DH, Deffenbacher JLJTCP. Stress inoculation training. 1988;16(1):69-90.

30. Sheehy R, Horan JJ. Effects of Stress Inoculation Training for 1st-Year Law Students. International Journal of Stress Management. 2004;11(1):41.

31. Hains AA, Ellmann SW. Stress inoculation training as a preventative intervention for high school youths. Journal of Cognitive Psychotherapy. 1994;8(3):219-32.

32. Szabo Z, Marian M. Stress inoculation training in adolescents: Classroom intervention benefits. Journal of Evidence-Based Psychotherapies. 2012;12(2):175.

33. Narimani M, Hasanzadeh S, Abolghasemi A. The effectiveness of stress inoculation training in decreasing stress, anxiety and depression of pre-university girl students. Journal of school psychology. 2012;1(3):101-17.

34. Palupi EPD, Sunawan S, Murtadho A. The Effectiveness of Group Counseling With Stress Inoculation Training to Improve Students Self-Esteem. Jurnal Bimbingan Konseling. 2020;9(3):164-8.

35. Serino S, Triberti S, Villani D, Cipresso P, Gaggioli A, Riva G. Toward a validation of cyber-interventions for stress disorders based on stress inoculation training: a systematic review. Virtual Reality. 2014;18(1):73-87.

36. Cohen S. Perceived stress in a probability sample of the United States. 1988.

37. Chen CH, Tseng YF, Chou FH, Wang SY. Effects of support group intervention in postnatally distressed women. A controlled study in Taiwan. Journal of psychosomatic research. 2000;49(6):395-9.

38. Yang TZ, Huang HT. An epidemiological study on stress among urban residents in social transition period. Zhonghua liu xing bing xue za zhi = Zhonghua liuxingbingxue zazhi. 2003;24(9):760-4.

39. Liu X, Zhao Y, Li J, Dai J, Wang X, Wang S. Factor Structure of the 10-Item Perceived Stress Scale and Measurement Invariance Across Genders Among Chinese Adolescents. Frontiers in psychology. 2020;11:537.

40. Mellor D, Vinet EV, Xu X, Hidayah Bt Mamat N, Richardson B, Román F. Factorial Invariance of the DASS-21 Among Adolescents in Four Countries. European journal of psychological assessment : official organ of the European Association of Psychological Assessment. 2015;31(2):138-42.

41. Lambert M, Hansen N, Umpress V, Lunnen K, Okiishi J, Burlingame G, et al. Administration and scoring manual for the OQ-45.2. 1996:50-80.

42. Miller SD, Duncan B, Brown J, Sparks J, Claud DJJobT. The outcome rating scale: A preliminary study of the reliability, validity, and feasibility of a brief visual analog measure. 2003;2(2):91-100.

43. She Z, Sun Q-w, Jiang G-rJCJoCP. Reliability and validity of Chinese version of Outcome rating scale. 2017.

44. Li C, Liu Q, Hu T, Jin X. Adapting the short form of the Coping Inventory for Stressful Situations into Chinese. Neuropsychiatric disease and treatment. 2017;13:1669-75.

45. Heber E, Ebert DD, Lehr D, Cuijpers P, Berking M, Nobis S, et al. The Benefit of Web- and Computer-Based Interventions for Stress: A Systematic Review and Meta-Analysis. Journal of medical Internet research. 2017;19(2):e32.

46. Plantinga L, Lim SS, Bowling CB, Drenkard C. Perceived stress and reported cognitive symptoms among Georgia patients with systemic lupus erythematosus. Lupus. 2017;26(10):1064-71.

47. Lu W, Bian Q, Wang W, Wu X, Wang Z, Zhao MJPo. Chinese version of the Perceived Stress Scale-10: A psychometric study in Chinese university students. 2017;12(12):e0189543.

48. Cohen S, Kamarck T, Mermelstein R. A global measure of perceived stress. Journal of health and social behavior. 1983;24(4):385-96.

49. Roberti JW, Harrington LN, Storch EAJJoCC. Further psychometric support for the 10‐item version of the perceived stress scale. 2006;9(2):135-47.

50. Andreou E, Alexopoulos EC, Lionis C, Varvogli L, Gnardellis C, Chrousos GP, et al. Perceived Stress Scale: reliability and validity study in Greece. International journal of environmental research and public health. 2011;8(8):3287-98.

51. Ng S-m. Validation of the 10-item Chinese perceived stress scale in elderly service workers: one-factor versus two-factor structure. BMC psychology. 2013;1(1):9.

52. Melnyk BM, Amaya M, Szalacha LA, Hoying J, Taylor T, Bowersox K. Feasibility, Acceptability, and Preliminary Effects of the COPE Online Cognitive-Behavioral Skill-Building Program on Mental Health Outcomes and Academic Performance in Freshmen College Students: A Randomized Controlled Pilot Study. J Child Adolesc Psychiatr Nurs. 2015;28(3):147-54.

53. Denovan A, Dagnall N, Dhingra K, Grogan SJSiHE. Evaluating the Perceived Stress Scale among UK university students: implications for stress measurement and management. 2019;44(1):120-33.

54. Kaya C, Tansey TN, Melekoglu M, Cakiroglu O, Chan F. Psychometric evaluation of Turkish version of the Perceived Stress Scale with Turkish college students. Journal of mental health (Abingdon, England). 2019;28(2):161-7.

55. Mitchell AM, Crane PA, Kim Y. Perceived stress in survivors of suicide: psychometric properties of the Perceived Stress Scale. Research in nursing & health. 2008;31(6):576-85.

56. Örücü MÇ, Demir AJS, Stress HJotISftIo. Psychometric evaluation of perceived stress scale for Turkish university students. 2009;25(1):103-9.

57. Perera MJ, Brintz CE, Birnbaum-Weitzman O, Penedo FJ, Gallo LC, Gonzalez P, et al. Factor structure of the Perceived Stress Scale-10 (PSS) across English and Spanish language responders in the HCHS/SOL Sociocultural Ancillary Study. Psychological assessment. 2017;29(3):320-8.

58. Baik SH, Fox RS, Mills SD, Roesch SC, Sadler GR, Klonoff EA, et al. Reliability and validity of the Perceived Stress Scale-10 in Hispanic Americans with English or Spanish language preference. Journal of health psychology. 2019;24(5):628-39.

59. Ranganathan P, Pramesh C, Aggarwal R. Common pitfalls in statistical analysis: intention-to-treat versus per-protocol analysis. Perspect Clin Res. 2016; 7 (3): 144–6.

# Appendices

## Appendix 1

The details of the intervention content

| **First phase:**  **Conceptualization** | **Aims:**   1. Build relationship with the participants 2. Introduce the online intervention: stress inoculation training, targeted mental health conditions; outcomes the participant may expect from the intervention 3. Introduction about confidentiality and privacy 4. Implement assessments 5. Analyse current problems   **Duration:**  30 minutes  **Techniques:**   1. Interview 2. Imagery recall 3. Self-monitoring 4. Behavioural assessment 5. Psychological testing | **Session 1:**  1. Introduction about the online intervention and ethical considerations;  2. implement assessments: PSS-10; DASS-21; FS-8; CISS-SFC  3. what is stress, coping strategies, emotions; and the transactional nature of psychological stress  **Session 2:**  Analyse current problem (nature of the participant’s stress and coping strategies)  **Session 3:**  Offer a conceptual model of the participant’s stress reactions |
| --- | --- | --- |
| **Second phase:**  **Skills acquisition and rehearsal phase** | **Aims:**  1. ensure that the participant develop the capacity to effectively execute coping responses  2. collect information about the participant’s attitudes and expectations for each particular training technique that is introduced.  3. nurture a flexible coping repertoire and to work with participants collaboratively, to select, test, and assess the merits of various coping procedures  4. teach the participant coping skills  **Duration:**  30 minutes  **Approaches:**  1. clinical interview  2. attuned ear for distorted thinking styles  3. evaluate the participant logically  4. use of 0-100 scale in the appraisals of stressful situations  5. Ellis ABC approach | **Session 4: relaxation skills training**  1. introduce the stress-tension cycle.  2. tap the participant’s previous experience with, concern with, and expectations concerning relaxation.  3. teach the participant relaxation skills through guided relaxation and deep breathing.  4. encourage the participant to apply the relaxation skills into daily life.  **Session 5: coping strategies-cognitive restructuring**  1. “thought catching”—help the participant become aware of automatic thoughts.  2. introduce common “thought distortions”: generalization, “must” or “should” statements, black and white thinking, catastrophizing, jump to conclusions, emotional thinking, ignore positive, personalization, predict the future, read others’ minds.  3. help the participant recognize that their thoughts are not usually the facts, but the hypotheses worthy of testing.  4. encourage participants to do personal experiments about their thoughts.  **Session 6 and 7: problem-solving training**  1. problem identification--define stressors or stress reactions as a problem to be solved.  2. goal selection—set realistic goals as concretely as possible by stating the problem in behavioural terms and by delineating steps necessary to reach each goal.  3. generation of alternatives—generate a range of possible alternative courses of action.  4. change of position—imagine how others might respond if asked to deal with similar stress problem.  5. Evaluation—evaluate the pros and cons of each proposed solution and rank order the solutions from least to most practical and desirable.  6. strategy rehearsal—rehearse strategies and behaviours by means of imagery, behavioural rehearsal, and graduated practice.  7. encourage and reward—expect failures but reward self for having tried.  **Session 8: self-instructional training, or guided self-dialogue**  1. Review with the participant the various stages if their .stress experience (i.e., preparation for stressors, confrontation, critical moments and self-reflection)  2. work with the participant to generate adaptive self-statements for 4 stages of stress experience (i.e., preparing for stressor; confronting and handling the stressor; coping with feelings of being overwhelmed; evaluation of coping efforts and self-rewards)  **Session 9: assertiveness**  1. Help participants understand what is assertiveness and the benefits of being assertive.  2. Help participants distinguish between passive, aggressive and assertive communication  3. Train participants with assertive techniques |
| **Third phase:**  **Application and follow-through** | **Aims:**  1. To encourage the participant to implement coping responses in day-to-day situations.  2. To maximize chances of generalized change  **Approaches:**  1. imagery rehearsal  2. behavioural rehearsal  3. role play  4. modelling  5. graduated in vivo practice | **Session 10: Imagery rehearsal**  1. The app help the participant with generating a hierarchy of scenes, from least to most stressful. Usually the length lasts in 1-5 minutes  2. The participant is encouraged to use any personally generated self-statements and images that would facilitate coping.  3.If the participant experience difficulty in reducing stress for any one scene, the app can either (i) go back to a less intensely stressful scene from the hierarchy or (ii) discuss with the participant about his/her “catastrophizing” self-statement when the participant become overwhelmed  4. Throughout the imagery rehearsal procedure, always check with the participant about his/her reactions to the imagery process, discussing the participant’s ability to visualize and when they can use coping techniques in vivo.  **Session 11: behavioural rehearsal, role play and modelling**  1. The app role play with the participant to practice coping skills (communication skills, mobilizing social support, and anger control)  2. The app shows short videos of others coping with stressful events, and discuss with the participant about other people’s coping efforts.  **Session 12: Relapse prevention and follow-through**  1. The app encourage participants to anticipate failures and setbacks  2. The app work with the participant to rehearse how they will respond to such lapses  3. The app can use spontaneous relapses that occur during the course of training as opportunities to develop resilience  4. help the participant to normalize setbacks, and educate the participant that our aim is not to eliminate stress, but use the stress constructively  5. The app convey to the participant that the “door is always open”, and encourage the participant to seek additional help if needed  6. Provide the participant with additional help resources the participant can seek when needed. |

## Appendix 2

Informed consent materials

**Consent Form**

**Research Title:** The feasibility, acceptability and efficacy of an online self-help intervention for stress

management among adolescents in China

**Researcher(s):**

Dr Sisira Edirippulige, Senior Lecturer and Course Co-ordinator, Centre for Online Health, Faculty of Medicine, The University of Queensland, Australia

Dr Matthew Bambling, Director of Post Graduate Mental Health, Faculty of Medicine, The University of Queensland; Research Fellow, Centre for Online Health, The University of Queensland. Australia

Prof Xuejun Bai, Academy of Psychology and Behavior, Tianjin Normal University, China

Ms Xiaoyun Zhou, PhD candidate, Centre for Online Health, The University of Queensland, Australia

Name of the participant: _________________________________

I consent to participate in this research project. It has been explained to me that the purpose of this research is to investigate The feasibility, acceptability and efficacy of an online self-help intervention for stress management among adolescents in China. I have also been provided with a written project information sheet in a language that I can understand.

The possible risks of participating in this research have been explained to my satisfaction. I understand that in this research I will be required to (i) download an app for stress management; (ii) use the app weekly for 12 weeks; (iii) my app usage information will be collected by the researcher; and (iv) take psychological assessment at three points via the website provided in the app.

I understand that my participation is voluntary and I am free to withdraw from this research anytime without needing to provide any explanation, and I would not receive any penalty or bias as a result of my withdrawal. Should I decide to withdraw, I understand that my data will be destroyed and will not be used in the research.

All information collected from this study will be treated as strictly confidential. All the data will be kept in a locked filing cabinet or on a password-protected computer in a secure office at the Centre for Online at the University of Queensland. All reporting about the research will be aggregated so that no individual can be identified. The information about the participants will be kept separately from the data, and only Xiaoyun Zhou, the investigator, will have access to the data. I understand that given the small number of participants, it is not possible to guarantee complete anonymity.

I consent for my data to be used in future research that is an extension of or related to this project.

I understand that this research adheres to the Guidelines of the ethical review process of The University of Queensland and the National Statement on Ethical Conduct in Human Research. I have been provided with contact details of the researcher, as well as UQ Ethics Coordinator.

Participant signature _______________________________Date_____________________________

Parent/guardian signature_______________________

relationship to the participant __________Date __________________________________________

Ethics ID number: [2021/HE000791](https://my-research.research.uq.edu.au/OmniNet/Application/EditApplication?applicationId=29874&applicationVersionId=30600)
