# Supplementary material for: The feasibility, acceptability and efficacy of an app-based intervention (the Coping Camp) in reducing stress among Chinese school adolescents: A cluster randomised controlled trial
Source: PLoS One. 2023 Nov 27;18(11):e0294119. doi: 10.1371/journal.pone.0294119 (PMC10681230; doi:10.1371/journal.pone.0294119)
Supplement: S2 File — (DOCX) [file pone.0294119.s002.docx]

**Tianjin Normal University Scientific Research Ethics Review Form**

Application Number (No. 2021041901)

| Project Title: The feasibility, acceptability and efficacy of an online self-help intervention for stress management among adolescents in school settings in China | |
| --- | --- |
| project type ： basic research Research duration: April 2021-December 2022 | |
| Project Manager： Xuejun Bai Title：Professor  Department (academy)：Academy of Psychology and behavior | |
| Cooperative research unit: University of Queensland, Australia Cooperative researcher: Xiaoyun Zhou (PhD student) Tel：+61 731768181 | |
| Request review type: Application project, project research after approval | |
| Review | Experimental program ： Meet the requirements √ non-compliant |
|  | Consent Form： Meet the requirements √ non-compliant |
| **Involving human-related research content and research program summary:**  In China, it is reported that 49% of high school students have obesity and stress-related health problems, while 60% of high school students lack sleep and sleep. Excessive stress can impair academic performance and is related to mental health disorders and suicidal ideation. In Western countries, some evidence shows that training students to learn stress coping skills is beneficial. Management interventions may benefit Chinese high school students by providing accessible and evidence-based interventions. Studying the potential of using online interventions to provide stress coping skills (stress injection training; SIT) among Chinese high school students. This study tests whether school-based online stress management interventions are feasible, feasible and effective in the context of Chinese high schools.  As part of the research, an online stress management program will be developed to provide SIT. Focus group interviews will be conducted to inform the design of the online plan. The secondary purpose of this study is to assess whether the online program (i) changes symptoms of worry and depression; (ii) affects operations; (iii) affects coping behavior; (iv) is feasible and acceptable.  The main outcome measure is the 10 items of the Sensory Stress Scale (PSS-10). Secondary outcome measures will be tools related to worry, depression, function, and stress coping behavior. Data will be collected at the replacement (T0), 12 weeks after the change (T1), and 16 weeks after the change (T2). It can be evaluated through statistics. It can be assessed by conducting focus group interviews. The effect of the intervention on the primary and secondary outcomes will be assessed through the use of appropriate treatment analysis and linear mixed models.  Research objects: high school students  Research method: two-arm randomized controlled trial | |
| **The applicant promises:**  I will carry out research activities in strict accordance with the relevant ethical requirements, and if there is any violation, I shall bear the responsibility.    Signature of person in charge: Xuejun Bai  Date: April 10, 2021 | |
| **Approval comments**  After review, the research protocol complies with general ethical principles, and it is agreed to carry out relevant research.  Chapter of the Ethics Committee of Tianjin Normal University  April 19, 2021 | |
