# Supplementary material for: The feasibility, acceptability and efficacy of an app-based intervention (the Coping Camp) in reducing stress among Chinese school adolescents: A cluster randomised controlled trial
Source: PLoS One. 2023 Nov 27;18(11):e0294119. doi: 10.1371/journal.pone.0294119 (PMC10681230; doi:10.1371/journal.pone.0294119)
Supplement: S3 File — (PDF) [file pone.0294119.s003.pdf]

THE UNIVERSITY OF QUEENSLAND
AUSTRALIA
CREATE CHANGE
Research Ethics and Integrity

Human Research Ethics Approval

Project Number: 2021/HE000791

Project Title: The feasibility, acceptability and efficacy of an online self-help intervention for stress management among adolescents in school settings in China

Version: 0.03

Chief Investigator: Dr Matthew Bambling

Centre for Health Services Research

Co-Investigator(s)
Prof Xuejun Bai
Dr Sisira Kumara Edirippulige
Ms Xiaoyun Zhou

Funding Body (UQ ref#):

Approving Committee: University of Queensland Human Research Ethics Committee B

Approval End Date: 14 Sep 2022

Date of Approval: Tuesday, 14 September 2021

University of Queensland Human Research Ethics Committee B confirms that this project meets the requirements of the National Statement on Ethical Conduct in Human Research (2007, current revision). The University's human research ethics committees are organised and operate in accordance with the National Statement on Ethical Conduct in Human Research (2007, current revision).

Approved Documents

Document Type	File Name	Document Tile	Application Version	Document Version	Last Modified
Change Tracking	2021_HE000791 v0_02 - v0_03 Changes.pdf	2021/HE000791 v0_02 - v0_03 Changes	0.3	1	9/09/2021 9:38:08 PM
Application Attachment	Study 3 Focus group interview questions.docx	According to the advice from Ethics Office, we applied the ethics for whole PhD, which includes a randomised controlled trial and a focus group interview, this document is for focus group interview questions.	0.2	1	9/09/2021 9:38:04 PM
Application Attachment	announcement.docx	announcement English	0.2	1	9/09/2021 9:38:02 PM
Application Attachment	announcement Chinese.docx	announcement, Chinese version	0.2	1	9/09/2021 9:38:02 PM
Application Attachment	Requested additional information 2021HE000791.docx	Answers to all requests sent by Ethics Office, you might need it to refer to while reviewing our application.	0.2	1	9/09/2021 9:38:03 PM
Application Attachment	Confirmation approval of PhD_Xiaoyun Zhou.pdf	Confirmation approval of PhD Xiaoyun Zhou	0.2	1	9/09/2021 9:38:02 PM
Application Attachment	flyer and advertisement Chinese.docx	Flyer and advertisement in Chinese, they are the same	0.2	1	9/09/2021 9:38:03 PM

Application Attachment	flyer and advertisement.docx	Flyer and advertisement in English, they are the same	0.2	1	9/09/2021 9:38:02 PM
Application Attachment	GKA_MDIS__EnglishTranslation.docx	Gate Keeper Approval_English Translation_Mianyang Donchen International School	0.2	1	9/09/2021 9:38:03 PM
Application Attachment	GKA_MNS_EnglishTranslation.docx	Gate Keeper Approval_English Translation_Mianyang Nanshan High School	0.2	1	9/09/2021 9:38:03 PM
Application Attachment	GKA_MKYZ_EnglishTranslation.docx	Gate Keeper Approval_English Translation_No.1 High School of Science City Sichuan	0.2	1	9/09/2021 9:38:03 PM
Application Attachment	GKA_MDIS.jpg	Gate Keeper Approval_Mianyang Donchen International School	0.2	1	9/09/2021 9:38:03 PM
Application Attachment	GKA_MNS.jpg	Gate Keeper Approval_Mianyang Nanshan High School	0.2	1	9/09/2021 9:38:03 PM
Application Attachment	GKA_MKYZ.jpg	Gate Keeper Approval_No.1 High School of Science City Sichuan	0.2	1	9/09/2021 9:38:03 PM
Application Attachment	Requested additional information for revised application.docx	NEW: this is requested additional information for the revised application.	0.3	1	9/09/2021 9:38:04 PM
Application	Output Form.pdf	Output Form	0.3	3	9/09/2021 9:38:01 PM
Application Attachment	ProjectDescriptionProtocol_updated.docx	Project Description	0.2	1	9/09/2021 9:38:04 PM
Application Attachment	Consent form_revised.docx	revised PCF according to the request from Ethics Office	0.2	1	9/09/2021 9:38:04 PM
Application Attachment	Participant Information Sheet_revision.docx	revised PIS according to the request from Ethics Office	0.2	1	9/09/2021 9:38:04 PM
Application Attachment	risk assessment_UQ student studying remotely.docx	risk assessment	0.2	1	9/09/2021 9:38:02 PM
Application Attachment	text messages sent to students.docx	text messages sent to participants for recruitment for the focus group interview which will happen after the completion of the RCT trial.	0.2	1	9/09/2021 9:38:04 PM

Ethics committee representative

Diann Eley
Acting Chair
University of Queensland Human Research Ethics Committee B EC00457
The University of Queensland
